# LSR overexpression induces chemoresistance in triple negative breast cancer cells through MDR1 upregulation and apoptosis attenuation

**DOI:** 10.1371/journal.pone.0336124

**Published:** 2025-11-03

**Authors:** Ming Zhao, Zhikun Ma, Amanda B. Parris, Xiaohe Yang

**Affiliations:** Department of Biological and Biomedical Sciences, Julius L. Chambers Biomedical/Biotechnology Research Institute, North Carolina Central University, Kannapolis, North Carolina, United States of America; Weill Cornell Medicine, UNITED STATES OF AMERICA

## Abstract

Chemoresistance in breast cancer therapy, especially for triple negative breast cancer (TNBC) remains a significant challenge. Recent studies showed that overexpression of lipolysis-stimulated lipoprotein receptor (LSR), known as a tricellular tight-junction protein, was detected in TNBC and MDR1 was among LSR upregulated genes in a screening assay but its functional impact has not been studied. This study aimed to characterize LSR overexpression-induced regulation of MDR1 in TNBC cells focusing on chemoresistance. LSR was overexpressed in MDA-MB-231 cells and knocked-out via CRISPR/Cas9 in MDA-MB-468 cells for functional studies. Chemoresistance of individual cell lines was evaluated with doxorubicin treatment, followed by cell proliferation, invasion, colony formation and apoptosis assays. Modulated protein and mRNA levels of specific genes were assessed with Western blotting and RT-qPCR. MDR1 inhibitor verapamil and MDR1-targeted siRNA were used to evaluate the functional impact of LSR-induced MDR1. Overexpression of LSR not only promotes cell proliferation and invasion in MDA-MB-231 cells, but also renders the cells resistant to doxorubicin. LSR induces MDR1 expression at both mRNA and protein levels. Moreover, inhibition of MDR1 with specific inhibitor verapamil or MDR1 knockdown reversed cellular resistance to doxorubicin in LSR-overexpressing MDA-MB-231 cells. In contrast, knockout of LSR expression in MDA-MB-468 cells, which express higher levels of LSR, significantly sensitized the cells to doxorubicin-induced growth inhibition and apoptosis. Our data demonstrated that LSR overexpression promotes TNBC cell proliferation and invasion, and upregulation of MDR1 in these cells renders them resistant to doxorubicin, suggesting that targeting LSR could be a useful strategy to overcome chemoresistance in TNBC.

## Introduction

Lipolysis-stimulated lipoprotein receptor (LSR) is a transmembrane protein crucial for the metabolism of triglyceride-rich lipoproteins, facilitating their endocytosis [1,2]. Loss of LSR in liver tissues induces systemic hyperlipidemia in mice [2]. Additionally, LSR plays a role in cell adhesion and tight junction formation [3]. Recent studies have highlighted LSR’s overexpression in various cancers, including breast, ovarian, and endometrial cancers [4]. Specifically, LSR expression is significantly increased in all breast cancer subtypes as compared to adjacent normal tissues, which is most evident in basal-like breast cancer [5]. Analysis of TCGA data suggests a significant association between LSR overexpression and breast cancer in African American women, indicating a potential link to breast cancer disparities [5]. Thus, investigating the impact of LSR overexpression on breast cancer development and treatment responses holds significant importance.

The association between LSR and breast cancer risk is also supported by studies using preclinical models. It was reported that overexpression of LSR in Hs578t cells, a claudin-low breast cancer cell line, enhances cell growth, invasion, and anchorage-independent growth [6]. Overexpression of LSR in basal-like breast cancer cells promotes xenograft tumor development [5]. Moreover, LSR enrichment has been observed in breast cancer stem-like cells (CD44^high^/CD24^low^) that were resistant to chemotherapy [7]. Recent findings indicate that LSR, traditionally recognized as a membrane protein, can translocate to the nucleus and act as a transcriptional regulator [5]. However, the target genes and effectors of LSR-mediated tumorigenesis remain poorly understood. Despite evidence suggesting that LSR signaling drives aggressive tumor-initiating cell behaviors, the role of LSR in therapeutic responses has been rarely reported.

The MDR1 gene encodes a protein that functions as an ATP-dependent drug efflux pump, contributing to multidrug resistance in cancer cells [8]. Upregulation of MDR1-associated chemoresistance poses a significant challenge in breast cancer treatment. Notably, dysregulation of MDR1 plays a crucial role in the evasion of therapeutic action by cancer stem cells [9]. While MDR1 was identified as a candidate gene in LSR-induced gene expression in a screening study [5], the functional connection remains to be established. Conversely, LSR has been reported to be overexpressed in CD44^high^/CD24^low^ breast cancer stem cells exhibiting distinctive chemoresistant properties [7]. Thus, elucidating the relationship between LSR and MDR1 and their roles in chemoresistance is of paramount importance.

In this study, we established triple-negative breast cancer cell line models with either LSR overexpression or knockout and investigated the impact of LSR on MDR1 expression and chemoresistance. Our findings demonstrate that LSR-induced upregulation of MDR1 plays a pivotal role in LSR-associated chemoresistance, offering novel insights into breast cancer treatment resistance.

## Materials and methods

### Reagents

Doxorubicin hydrochloride was from Pfizer Inc. (New York, USA). Verapamil (S4202) was from Selleck Chemicals (Houston, USA). Primary antibodies MDR1(1:500; sc-55510) and β-Actin (1:1000; sc-47778) were from Santa Cruz Biotechnology (Dallas, USA). PARP antibody (1:2000; Cat:9546), Caspase 3 antibody (1:2000; Cat:9662), Cleaved Caspase 3 antibody (1:2000; Cat:9664) and LSR antibody (1:1000; Cat:14804) were from Cell Signaling Technology (Danvers, USA). Ki-67 antibody (1:1000; Cat:PA5–19462) was from Thermo Fisher Scientific (Waltham, USA).

### Cell culture and the establishment of LSR overexpressing or knockout sublines

MDA-MB-231 and MDA-MB-468 TNBC cell lines were purchased from American Type Culture Collection (ATCC, Manassas, USA). The cells were cultured in DMEM/F-12 medium supplemented with 10% fetal bovine serum (FBS), penicillin (100 U/ml), and streptomycin (100 μg/ml). To establish stable LSR-expressing MDA-MB-231 cells (231/LSR), the cells were infected with control and LSR-encoding lentivirus, and then selected by puromycin, followed by verification of pooled clones. For the establishment of MDA-MB-468 cells with LSR knockout (468/LSRKO), MDA-MB-468/cas9 cells were infected with control and LSR sgRNA encoding lentivirus. For lentivirus packaging, 293T cells were transfected with a lentivirus packing kit (Genecopoeia, Rockville, USA) along with EX-NEG-Lv105 (control), EX-L5381-Lv105 (LSR-expressing) and HCP304262-LvSG03−3 (knockout of LSR) plasmids, respectively as in our previous reports [10].

### Luciferase assay

Cells were seeded into 12-well plates at a density of 1 x 10^4^ cells per well. After 24 hours of incubation, cells were co-transfected with plasmids encoding the MDR1 promoter-firefly-luciferase (pMDR1–1202, Cat: 37627, Addgene) and the SV40 promoter-Renilla luciferase with luciferase construct (pRL-SV40, Cat: 27163, Addgene) using EndoFectin Transfection Reagents (Cat: EF013, GeneCopoeia). After 48 hours, luciferase activities were measured using the Dual-Luciferase Assay System (Cat: E1910, Promega) according to the manufacturer’s instructions. The relative MDR1-firefly luciferase activities were normalized to the corresponding Renilla luciferase activities. Data from triplicate samples were analyzed using Student’s *t*-test.

### Immunofluorescence (IF)

Cells cultured on coverslips were washed with PBS and fixed in 4% paraformaldehyde for 15 min at room temperature, followed by permeabilization with 0.1% Triton X-100 for 20 min and blocked with 3% BSA in PBST for 30 min. Primary antibodies diluted in 3% BSA (PBST) were applied overnight at 4°C. After three washes, Alexa Fluor 546-conjugated secondary antibodies (Invitrogen) diluted in 3% BSA (PBST) were added, followed by a 1 h incubation at room temperature in the dark. After additional washes, cells were mounted with Antifade Mounting Medium containing DAPI (Vector Labs). Fluorescence images were captured using a Nikon microscope.

### siRNA transfection

231/LSR cells were seeded in 6-well plates at a density of 2 × 10⁵ cells per well, 16 hours prior to transfection. Cells were transfected with either MDR1-specific siRNA SMART Pool (L-003868-00-0005) or non-targeting control siRNA (D-001810-01-05) (Horizon Discovery, Boyertown, PA) using DharmaFECT 1 transfection reagent (T-2001-01) according to the manufacturer’s instructions. Briefly, 5 μl of 10 μM siRNA and 5 μl of DharmaFECT reagent were each diluted in 190 μl of Opti-MEM (Gibco) and incubated separately for 5 minutes at room temperature. The two solutions were then combined, incubated for an additional 20 minutes, and added to the cells. At 48 hours post-transfection, cells were treated with doxorubicin and subjected to either CCK-8 assay or Western blot analysis, as appropriate.

### Cell viability assessment

The cells to be tested were inoculated into 96-well plates (1 × 10^3^ cells per well) for 24 h prior to treatment. The cells were then treated with indicated reagents at different concentrations for 96 h. Cell Counting Kit-8 (CCK-8; Enzo, Farmingdale, USA) was used to access cell viability at the endpoint. To this end, CCK-8 at 10 μl/well was added to the wells and incubated for 2 h. The cell survival fractions were calculated based on absorbance (A) at 450 nm measured with a microplate reader (SynergyMx, BioTek) [11], and the data was processed using GraphPad Prism 8 software. For the IC_50_ evaluation, the survival fractions of drug-treated cells were measured in three independent experiments. The statistical differences in IC_50_ values between the paired groups were analyzed using Welch’s t-test.

### Cell cycle analysis

Control and treated cells were collected and washed with PBS. The cells were fixed drop-wise with 70% ethanol and stored at −20°C overnight. The samples were then washed with ice-cold PBS twice, followed by incubation with the staining buffer (PI 33 μg/ml, 0.1% Triton X-100, 500 μg/ml RNase A) for 1 hour. The cells were then analyzed using a Guava EasyCyte 8 flow cytometer. The DNA content and cell cycle phases of individual samples were analyzed with ModFit LT Software [10].

### Colony formation assay

Cells were inoculated into 6-well plates at 600 cells/well and treated with specified conditions for 10 d. The medium was changed once on the third day during culturing. At the endpoint, the cells were washed with PBS twice and fixed with acetone/methanol (1:1) for 5 min. After staining with 0.5% crystal violet for 20 min, the plates were washed with water and air-dried. Images were captured with a Nikon stereoscopic microscope (SMZ-745T) and analyzed with ImageJ software.

### Cell invasion assay

A Biocoat Matrigel Invasion Chamber kit (BD Biosciences, Franklin Lakes, USA) was used to evaluate the invasion capability of the treated cells. Briefly, 2 × 10^4^ cells were seeded into the upper matrigel-coated chamber containing serum-free media. The lower chamber contained 600 μl of DMEM/10% FBS medium. After 24 h, the invaded cells were fixed with methanol and stained with 0.2% crystal violet. Images were captured using a Nikon SMZ 745T microscope. The number of invading cells was counted in five representative microscopic fields. The data were based on three independent experiments.

### Western blotting

The treated cells were collected and lysed in Laemmli sample buffer (Bio-Rad, Hercules, CA), followed by protein concentration determination with a BCA assay kit (Thermo Fisher Scientific, Waltham, USA). Thirty (30) μg of protein lysate from each sample was loaded for separation by SDS-PAGE electrophoresis. Separated proteins were transferred to polyvinylidene difluoride (PVDF) membranes. The blots were blocked with 5% non-fat milk in TBST and probed with specific primary antibodies at appropriate dilutions at 4°C overnight. After washing with TBST, the membranes were incubated with horseradish peroxidase-labeled secondary antibody (1:2000) for 1 h. The specific proteins were detected with chemiluminescence using the Super Signal West Dura Detection System (Thermo Fisher Scientific) and visualized by an Azure Biosystems Imager (Dublin, USA). The intensity of specific protein bands was quantified and normalized to the loading control (β-actin) using ImageJ software. The relative intensity of individual bands is indicated beneath the corresponding blots.

### Quantitative real-time PCR

mRNA levels of MDR1 were detected with quantitative real-time PCR (*q*-PCR) as previously described [12]. Total RNA was isolated with TRIzol Reagent. iScript cDNA Synthesis kit (Bio-Rad) was used for reverse transcription. SYBR green-based *q*-PCR assays were performed in triplicate using a Bio-Rad CFX96 PCR machine. The primers for MDR1 and GAPDH in the *q*-PCR assay are: MDR1, F: 5’-cccatcattgcaatagcagg-3’, R:5’-tgttcaaacttctgctcctga-3’; GAPDH, F: 5’-ctgacttcaacagcgacacc-3’, R: 5’-gtggtccaggggtcttactc-3’. The comparative-Ct method (ΔΔCt method) was used to calculate relative mRNA levels of individual samples.

### Cell death detection with ELISA

Cell Death Detection ELISA kit (Roche Life Science, Indianapolis, USA) was used for apoptosis assessment. The treated cells were collected and counted at the endpoint. Cell lysates were prepared by lysing 1 × 10^4^ cells of each sample in 200 μl buffer after a 30-min incubation. Twenty (20) μl of supernatant was transferred into a microplate for the reaction with the immunoreagents. The washed wells were incubated with substrate for color development, followed by absorbance reading at 405 nm using a microplate reader. Relative apoptosis based on triplicate samples was presented as a ratio of the apoptosis in drug treated groups over the control group.

### Statistical analysis

Quantitative results were statistically analyzed using GraphPad Prism 8 software. Comparisons between two groups in single-factor experiments were performed using a two-tailed Student’s t-test. IC₅₀ values from three independent experiments were analyzed using Welch’s t-test. Results involving multiple comparisons, such as clonogenic and apoptosis ELISA assays, were analyzed using one-way ANOVA followed by Tukey’s post hoc test. * *p* < 0.05; ** *p* < 0.01.

## Results

### LSR overexpression promotes cell proliferation and invasion in MDA-MB-231 cells

**Fig 1:**
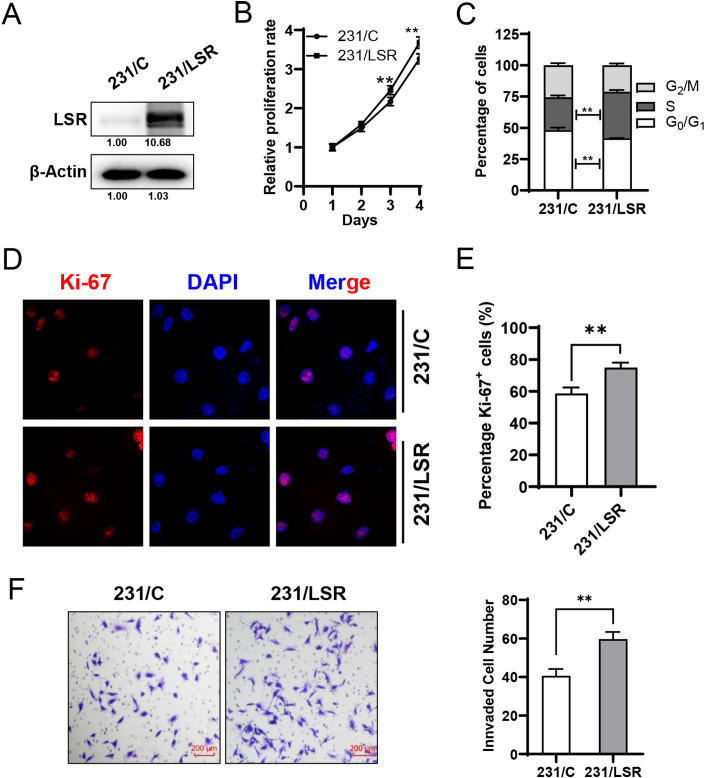
LSR overexpression promotes cell proliferation and invasion in MDA-MB-231 cells. **A)** Protein levels of LSR in control (231/C) and LSR overexpressing (231/LSR) MDA-MB-231 cells. **B)** LSR overexpression promotes cell proliferation, which was assessed with CCK-8 assay on 231/C and 231/LSR cells. Data represent mean ± SEM from three independent experiments. **C)** LSR overexpression induces a higher percentage of S phase cells in MDA-MB-231 cells. Cell cycle distribution of 231/C and 231/LSR cells was analyzed with Flow Cytometry (n = 3 per group). **D&E)** Immunofluorescence staining of Ki67 in 231/C and 231/LSR cells. **D.** Representative images of Ki67 (red) and nuclear staining with DAPI (blue). The percentage of Ki67 + cells in each subline was quantified in E. **F)** 231/LSR cells were more invasive than 231/C cells based on Transwell invasion chamber assays, as indicated by representative images and quantitative summary. **p < 0.01 by student’s t-test.

In our preliminary studies, we examined the expression of LSR in a group of commonly used breast cancer cell lines (S1 Fig). Between the two TNBC cell lines in the panel, we found that LSR levels were higher in MDA-MB-468 cells but lower in MDA-MB-231 cells. To study the role of LSR in TNBC cells, we established control and LSR overexpressing sublines of MDA-MB-231 cells, 231/C and 231/LSR, using lentiviral expression system (Fig 1A). Functional analysis demonstrated that LSR overexpression in the MDA-MB-231 cells promoted cell proliferation, as indicated by higher proliferation rate in CCK-8 assays and increased cell percentage in the S phase of cell cycle (Fig 1B and C). To confirm LSR overexpression-induced cell proliferation detected with cell cycle analysis, we examined Ki67 positive cells in 231/C and 231/LSR cells with immunofluorescence staining. The results indicate that the percentage of Ki67 + cells in 231/LSR cells was significantly higher than that of 231/C cells (Fig 1D and E). We further demonstrated that LSR overexpression in MDA-MB-231 cells also promoted cell invasion based on transwell invasion chamber assays (Fig 1F). While establishing LSR specific cell line models for further studies, these data also provide additional evidence supporting LSR-induced proliferation and invasion in TNBC cells for the following analysis.

### LSR overexpression renders MDA-MB-231 cells resistant to doxorubicin through inhibition of apoptosis

**Fig 2:**
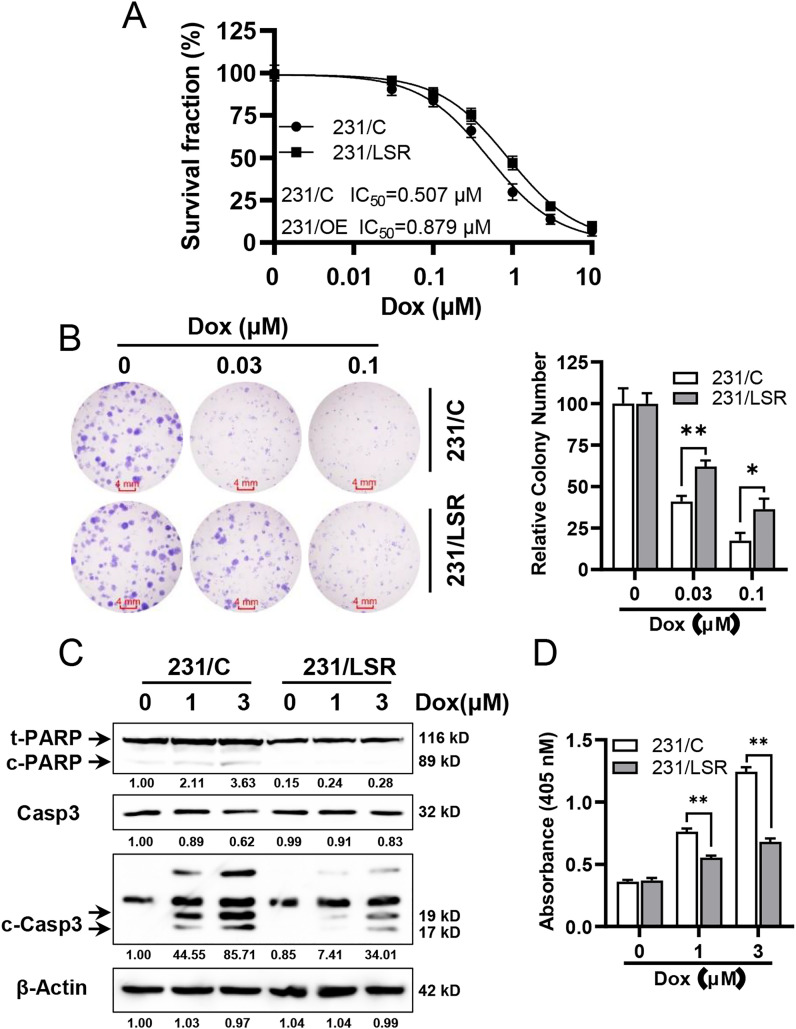
LSR overexpression renders MDA-MB-231 cells resistant to doxorubicin through inhibition of apoptosis. **A)** 231/LSR cells were more resistant to doxorubicin than 231/C cells. The paired cell lines were treated with doxorubicin with concentrations ranging from 0.03 to 10 µM for four days, followed by IC_50_ analysis based on CCK-8 assays. The statistical differences in IC_50_ values between the paired groups based on three independent experiments were analyzed using Welch’s t-test, with a significance level of *p* < 0.01. **B)** Clonogenic analysis of doxorubicin treated 231/C and 231/LSR cells. The cells were treated with doxorubicin at indicated concentrations for 10 d, followed by crystal violet staining. Colony numbers of each group were quantified and analyzed with Student’s *t*-*t*est. **C)** LSR overexpression attenuates doxorubicin-induced activation of Caspase cascade in MDA-MB-231 cells. The paired cell lines were treated with doxorubicin at the indicated concentrations for 24 hours, followed by Western blot detection of PARP (both total and cleaved PARP (c-PARP)), total Caspase 3 (Casp3), and cleaved Caspase 3 (c-Casp3). The relative protein levels of c-PARP, Casp3, and c-Casp3 (signals from both 19 and 17 kD bands) were quantified and are indicated beneath the corresponding blots. **D)** The effect of LSR overexpression on doxorubicin-induced apoptosis in MDA-MB-231 cells was measured using an apoptosis ELISA assay. Data from clonogenic assays and apoptosis ELISA were analyzed with one-way ANOVA followed by Tukey’s post hoc test, *p < 0.05; **p < 0.01.

To investigate the effect of LSR overexpression on the responses to chemotherapeutics, we examined the sensitivity of 231/C and 231/LSR cells to doxorubicin and cisplatin, which are commonly used chemotherapeutic agents. As shown in Fig 2A, the IC_50_ of doxorubicin for 231/C and 231/LSR cells were 0.507 µM (95% CI: 0.398 to 0.647 µM) and 0.879 µM (95% CI: 0.732 to 1.058 µM), respectively. The statistical differences in IC_50_ values of the two groups were very significant (*p* < 0.01) based on Welch’s t-test. Consistently, LSR overexpression also induced MDA-MB-231 cells resistant to cisplatin (S2A Fig), suggesting that LSR-associated chemoresistance is not limited to specific chemotherapy drugs. Given that both doxorubicin and cisplatin exhibited similar resistance patterns in 231/LSR cells, we chose to focus on doxorubicin in subsequent experiments. Doxorubicin was selected due to its well-established role in the study of chemoresistance mechanisms, allowing for a more in-depth exploration of LSR’s contribution to drug resistance in a more controlled setting. Next, we examined the effect of LSR overexpression on colony formation in doxorubicin-treated 231/C and 231/LSR cells. The results demonstrated that colony numbers in doxorubicin-treated 231/LSR cells were significantly higher than the control cells under the same conditions (Fig 2B). Data from both assays demonstrated that LSR overexpression renders the cells more resistant to doxorubicin. Aberrant apoptosis is commonly associated with chemoresistance. To understand the mechanism of LSR-mediated chemoresistance, we examined doxorubicin-induced apoptosis in control and LSR overexpressing cells. We first detected the cleavage of Caspase 3 and PARP, which are well-established markers for the activation of Caspase cascade and apoptosis [13]. As shown in Fig 2C, the levels of cleaved PARP (p89/c-PARP) and cleaved Caspase 3 (p19 and p17) were significantly decreased in doxorubicin treated 231/LSR cells as compared to the control cells, indicating inhibited apoptosis in 231/LSR cells. Data from apoptosis ELISA assays on doxorubicin treated 231/C and 231/LSR cells further confirmed the differential apoptotic responses between the two sublines (Fig 2D), as indicated by significantly reduced apoptosis in the treated 231/LSR cells. Taken together, these results demonstrated that LSR overexpression in MDA-MB-231 cells induces doxorubicin resistance through inhibition of apoptosis.

### MDR1 upregulation contributes to LSR-induced resistance to doxorubicin in MDA-MB-231 cells

**Fig 3:**
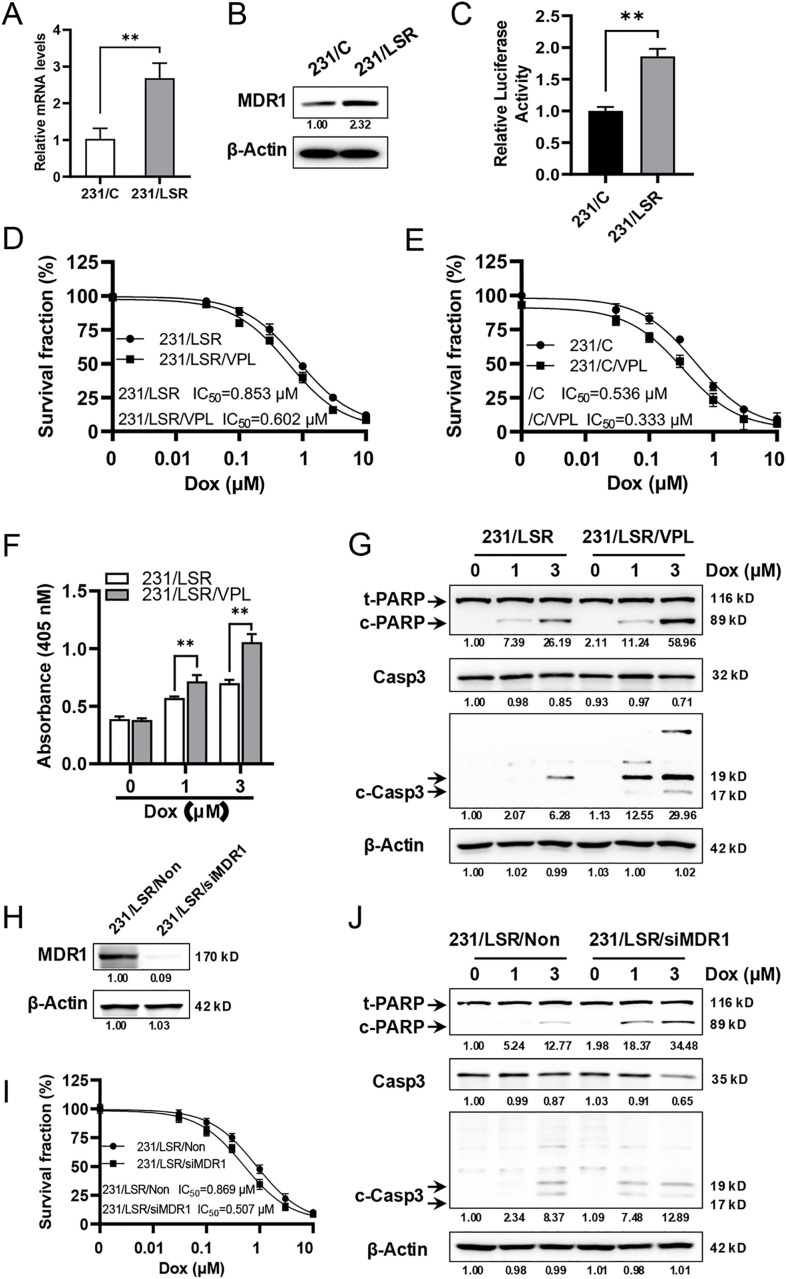
MDR1 upregulation contributes to LSR-induced resistance to doxorubicin in MDA-MB-231 cells. **A)** The mRNA levels of MDR1 in 231/C and 231/LSR cells were determined by *q*-PCR analysis. **B)** The protein levels of MDR1 in 231/C and 231/LSR cells were detected with Western blot. **C)** LSR overexpression in MDA-MB-231 cells induces MDR1 promoter-mediated luciferase activity. 231/C and 231/LSR cells were co-transfected with plasmids encoding MDR1 promoter-firefly-luciferase and SV40 promoter-Renilla luciferase, followed by luciferase analysis after 48 hours. The relative MDR1-luciferase activity for each cell line was normalized to Renilla-luciferase signals. **D & E)** MDR1 inhibitor verapamil sensitized 231/LSR (D) and 231/C (E) cells to doxorubicin. The cells were treated with doxorubicin at indicated concentrations in the absence or presence of 10 μM verapamil for four days, followed by survival fraction detection with CCK-8 assays. The IC_50_ of each group was calculated with GraphPad software. **F)** Inhibition of MDR1 enhances doxorubicin-induced apoptosis in 231/LSR cells measured by apoptosis ELISA. The cells were treated with doxorubicin in the presence or absence of verapamil for 24 hours, followed by the measurement of apoptotic substrates. **G)** Western blot detection of PARP (both total and cleaved PARP (c-PARP)), total Caspase 3 (Casp3), and cleaved Caspase 3 (c-Casp3) in 231/LSR cells treated with doxorubicin and/or verapamil as above. The relative protein levels of c-PARP, Casp3, and c-Casp3 (signals from both 19 and 17 kD bands) were quantified and are indicated beneath the corresponding blots. **(H–J)** MDR1 knockdown restores the sensitivity of 231/LSR cells to doxorubicin. Cells were transfected with control siRNA (231/LSR/Non) or MDR1-specific siRNA (231/LSR/siMDR1) for 48 h, followed by treatment with doxorubicin at the indicated concentrations. **(H)** Protein levels of MDR1 in control and siMDR1-transfected 231/LSR cells. **(I)** IC_50_ values of 231/LSR/Non and 231/LSR/siMDR1 were determined by CCK-8 assay after 4 days of treatment. **(J)** Western blot analysis of total and cleaved PARP (c-PARP) and Caspase-3 (Casp3, c-Casp3) after 24 h of treatment. Relative protein levels were quantified. Data from (A) and (C) were analyzed using Student’s t-test; IC_50_ values from **(D)**, (E) and (I) were analyzed using Welch’s t-test; data from (F) were analyzed using one-way ANOVA followed by Tukey’s post hoc test. **p < 0.01.

Multidrug resistance 1 (MDR1) gene encodes a membrane protein that can pump chemo-drugs out of cells and is associated with chemo-resistance [8]. A previous report indicated that LSR expression is enriched in cells with breast cancer stem cell phenotypes, which was associated with the upregulation of a set of genes, including MDR1, as determined with ChIP-Seq assays without functional characterization [5]. Given the critical role of MDR1 in chemoresistance, we focused on the connection between LSR overexpression and MDR1 to understand LSR-induced pathogenesis. To study the role of MDR1 in LSR overexpression-induced chemo-resistance, we examined MDR1 expression in control and LSR overexpressing MDA-MB-231 cells. The results showed that both mRNA and proteins levels of MDR1 were significantly increased in LSR overexpressing cells (Fig 3A and B), suggesting LSR overexpression induced MDR1 upregulation, which may contribute to chemoresistance. To demonstrate that LSR induces MDR1 upregulation through the induction of MDR1 gene transcription, we assessed MDR1-promoter-mediated transcription/expression of luciferase in 231/C and 231/LSR cells (Fig 3C). Increased MDR1-promoter-driven luciferase activity in 231/LSR cells, compared to controls, indicates that LSR induces MDR1 transcription. Condensed staining of LSR in the nucleus of 231/C cells and, in particular, 231/LSR cells (S3 Fig) suggests LSR’s active involvement in the regulation of nuclear activities. To determine the functional role of MDR1 in LSR-associated resistance, we tested the effect of combining doxorubicin with verapamil, an established MDR1 inhibitor [14,15], on the sensitivity of 231/LSR cells. We demonstrated that verapamil significantly attenuated LSR-associated doxorubicin resistance in MDA-MB-231 cells (Fig 3D). Possibly by acting on endogenous MDR1, the doxorubicin-verapamil combination also sensitized 231/C cells (Fig 3E). Focusing on 231/LSR cells, we further demonstrated that inhibition of MDR1 by verapamil resulted in increased apoptosis of 231/LSR cells, as indicated by apoptosis ELISA assays and the cleavage of PARP and Caspase-3 proteins (Fig 3F and G). Consistently, siRNA-mediated MDR1 knockdown (Fig 3H) restored doxorubicin sensitivity, as evidenced by reduced cell viability in CCK-8 assays and increased apoptotic markers on Western blot (Fig 3I, J). Collectively, these results demonstrated that MDR1 upregulation plays a critical role in LSR-mediated cell resistance to doxorubicin.

### LSR knockout sensitizes MDA-MB-468 cells to doxorubicin through induction of apoptosis

**Fig 4:**
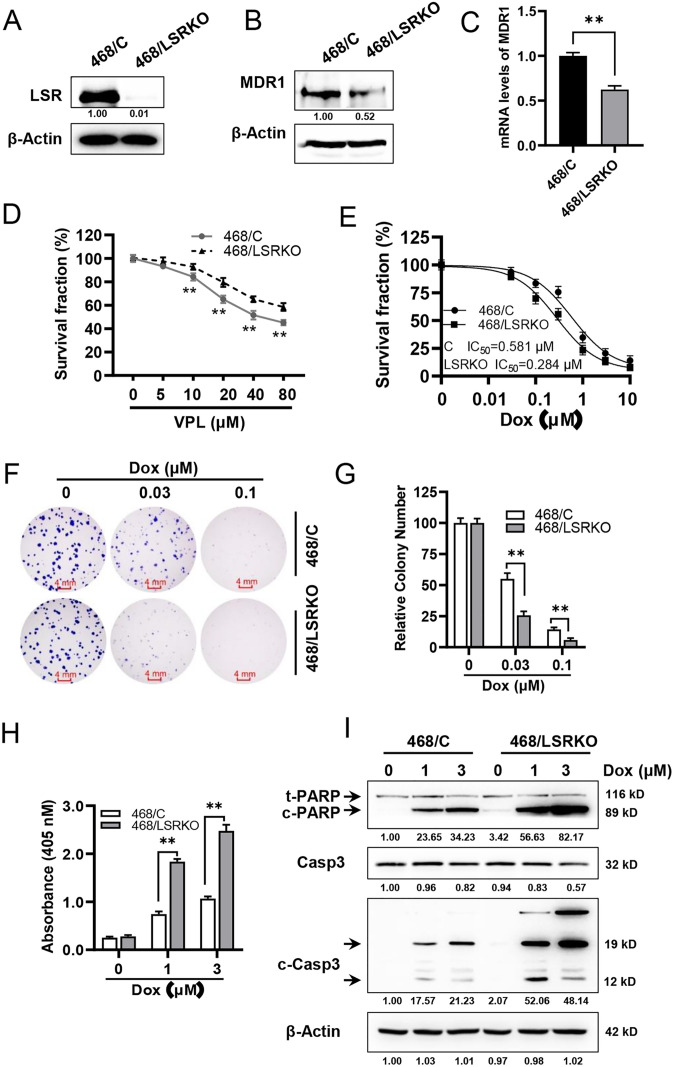
LSR knockout sensitizes MDA-MB-468 cells to doxorubicin through induction of apoptosis. **A)** Protein levels of LSR in control (468/C) and LSR knockout (468/LSRKO) sublines of MDA-MB-468 cells. **B)** Protein levels of MDR1 in 468/C and 468/LSRKO cells detected with Western blot and quantification of relative signals. **C)** mRNA levels of MDR1 468/C and 468/LSRKO cells detected with RT-qPCR. **D)** LSR knockout-induced MDR1 downregulation renders MDA-MB-468 cells resistant to verapamil. The paired cell lines were treated with verapamil (5, 10, 20, 40, 80 μM) for four days followed by CCK-8 assays. **E)** IC_50_ determination of 468/C and 468/LSRKO cells treated with doxorubicin. The paired cell lines were treated with doxorubicin at indicated concentrations for four days followed by IC_50_ analysis based on CCK-8 assays. **F & G)** Clonogenic assays of 468/C and 468/LSRKO cells treated with doxorubicin. The cells were treated with doxorubicin at indicated concentrations for 10 days, followed by crystal violet staining and the quantification of colony numbers. **H)** LSR knockout sensitizes doxorubicin-induced apoptosis in MDA-MB-468 cells measured with apoptosis ELISA assays. **I)** LSR knockout promotes doxorubicin-induced activation of Caspase cascade in MDA-MB-468 cells. 468/C and 468/LSRKO cells were treated with doxorubicin at indicated concentrations for 24 hours, followed by Western blot detection of PARP (both total and cleaved PARP (c-PARP)), total Caspase 3 (Casp3), and cleaved Caspase 3 (c-Casp3) in 231/LSR cells treated with doxorubicin and/or verapamil as above. The relative protein levels of c-PARP, Casp3, and c-Casp3 (signals from both 19 and 17 kD bands) were quantified and are indicated beneath the corresponding blots. Data from (C) were analyzed using Student’s t-test; IC50 values from (E) were analyzed using Welch’s t-test; data from **(D)**, (F) and (H)were analyzed using one-way ANOVA followed by Tukey’s post hoc test. **p < 0.01.

Results from MDA-MB-231 cells indicate that overexpression of LSR induces doxorubicin resistance. To confirm these findings, we examined the effect of LSR knockout on chemo-sensitivity in MDA-MB-468 cells, which is a basal-like TNBC cell line with higher levels of endogenous LSR (S1 Fig). Using lentivirus-mediated CRISPR-Cas9 technology, we generated stable sublines of control (468/C) and LSR knockout (468/LSRKO) MDA-MB-468 cells (Fig 4A). Examination of MDR1 expression in 468/C and 468/LSRKO cells indicated that the expression of MDR1 was significantly downregulated at both protein and mRNA levels (Fig 4B and C), supporting the connection between LSR and MDR1 expression. When the paired sublines were treated with MDR1 inhibitor, verapamil, the results showed that 468/LSRKO cells were more resistant to verapamil (Fig 4D), suggesting a correlation between LSR levels and MDR1-associated responses to chemotherapy. To test the effect of LSR knockout on the sensitivity of MDA-MB-468 cells to doxorubicin, we evaluated the IC_50s_ of doxorubicin-treated 468/C and 468/LSRKO cells. The results showed that LSR knockout significantly sensitized these cells to doxorubicin. The IC_50_s for 468/C and 468/LSRKO cells were 0.581 µM and 0.284 µM, respectively (Fig 4E). Consistently, LSR knockout also significantly sensitized MDA-MB-468 cells to cisplatin (S2B Fig). Moreover, results from the clonogenic assay further indicated that 468/LSRKO cells were more sensitive to doxorubicin than 468/C cells under the given conditions (Fig 4F and G). Moreover, analysis of apoptosis in the paired cell lines treated with doxorubicin demonstrated that the drug induced enhanced apoptosis in 468/LSRKO cells as compared to 468/C cells, as indicated by apoptosis ELISA data and increased cleavage of PARP and Caspase 3 in doxorubicin treated 468/LSRKO cells (Fig 4H and I). Of note, doxorubicin induced distinctive patterns of PARP and Caspase 3 cleavage (Fig 4I). The major cleaved peptides of cleaved Caspase 3 in these cells appear to p19 and p12 subunits. Taken together, data from MDA-MB-468 cells, which are from a different TNBC cell line using a different approach (LSR knockout), provide additional evidence supporting the role of LSR overexpression in chemoresistance in TNBC cells.

## Discussion

In this study, we established isogenic TNBC cell lines with LSR overexpression and characterized the effect of LSR dysregulation on tumor cell aggressiveness and therapeutic responsiveness. Our results highlight LSR’s implication in tumorigenesis through enhanced cell proliferation, invasion and notably, chemoresistance to doxorubicin and cisplatin. Our findings extend previous research on LSR’s function beyond its established role in cell adhesion/junction and lipoprotein endocytosis [2], underscoring its role in cancer biology and therapeutic responses. Since LSR overexpression is more frequently detected in TNBC and basal-like breast cancer in particular [5], results from our models based on basal/TNBC cell lines could be a valuable reference for relevant studies.

The effect of LSR on therapeutic resistance has been rarely reported, although a previous report detected LSR overexpression in chemoresistant breast cancer cells with cancer stem cell properties in a ChIP-Seq assay without mechanistic and functional verification [7]. Our results in this study indicate that LSR overexpression in MDA-MB-231 cells induces cancer cells resistant to doxorubicin and cisplatin (S2 Fig). In contrast, LSR knockout in MDA-MB-468 cells sensitized the cells to doxorubicin. Consistent results from MDA-MB-231 cells with LSR overexpression and MDA-MB-468 cells with LSR knockout provide additional support for the role of LSR in mediating chemoresistance. This inverse relationship between LSR expression and chemosensitivity in two distinct TNBC cell lines underscores the potential of LSR as a therapeutic target. Given the challenges of chemoresistance in TNBC treatment, these findings support targeting LSR as a novel approach to enhance the efficacy of chemotherapy in TNBC. Notably, a novel antibody therapy targeting LSR has been developed for ovarian cancer treatment [16]. Our results pave the way for further testing of LSR targeting drugs on TNBC tumors.

Our study also demonstrates LSR’s role in diminishing apoptosis, a fundamental response to chemotherapeutic agent [17], which underscores the complex interplay between LSR and cancer cell survival pathways. The attenuation of doxorubicin-induced apoptosis in LSR overexpressing cells, in context with the increased expression of MDR1, provides a potential link between LSR overexpression and chemoresistance, suggesting a dual mechanism of action that involves both evasion of cell death and active drug efflux. However, whether apoptotic response is merely an effect secondary to LSR-induced MDR1 upregulation or involves interactions between LSR and apoptotic machinery warrants further investigation.

A previous study using ChIP-Seq screening suggests that MDR1, along with a list of genes, could be regulated by LSR [5]. Our results support the association between LSR overexpression and upregulation of MDR1, which is of particular significance. MDR1, known for its role in drug efflux and chemoresistance [18], appears to be a key factor in LSR-mediated doxorubicin resistance. The significant increase in mRNA and protein levels of MDR1 in LSR-overexpressing MDA-MB-231 cells and its downregulation in LSR-KO MDA-MB-468 cells suggest a regulatory connection. Furthermore, the attenuation of chemoresistance by verapamil, an MDR1 inhibitor, in MDA-MB-231/LSR cells, provides functional evidence for this association. Further supporting the pivotal role of MDR1 in LSR-mediated chemoresistance, our siRNA-mediated knockdown experiments demonstrated that direct suppression of MDR1 expression restored doxorubicin sensitivity in LSR-overexpressing MDA-MB-231 cells. Reduction of MDR1 protein levels markedly decreased cell viability and reactivated the apoptotic response, as evidenced by enhanced PARP and Caspase-3 cleavage (Fig 3H-J). These findings confirm that MDR1 is a functional downstream effector of LSR, mediating drug resistance through both efflux-dependent and apoptosis-attenuating mechanisms. Together with the verapamil inhibition results, this provides compelling evidence that targeting MDR1 can effectively counteract LSR-induced chemoresistance in TNBC cells. However, the molecular mechanism of LSR-mediated upregulation of MDR1 requires further studies. Nevertheless, our findings not only highlight a novel mechanistic pathway in chemoresistance but also suggest potential therapeutic targets to overcome drug resistance in TNBC.

In summary, the present study elucidates the multifaceted role of LSR in TNBC, particularly highlighting its contributions to tumor aggressiveness and chemoresistance. These findings underscore LSR’s role in therapeutic responsiveness and suggest that targeting LSR, directly or through its downstream effectors like MDR1, could be a promising strategy in TNBC treatment. The work also lays a foundation for future studies on unraveling the detailed molecular mechanisms behind LSR-mediated effects and exploring the therapeutic potential of LSR inhibition in TNBC.

## Supporting information

S1 Fig LSR expression in breast cancer cell lines. Protein lysates of indicated cell lines were prepared. Protein levels of LSR were detected with Western blotting and the relative LSR intensity was quantified.
(TIF)

S2 Fig LSR expression is associated with cisplatin sensitivity in MDA-MB-231 and MDA-MB-468 cells. **A & B)** LSR overexpressing MDA-MB-231 cells (231/LSR), LSR knockout MDA-MB-468 cells (468/LSRKO) and corresponding control cells were seeded into 96-well plates at a density of 1000 cells/well. After 24 h of incubation, the cells were treated with different concentrations of cisplatin (0, 0.03, 0.1, 0.3, 1, 3 μM) for four days. The cell survival was evaluated by CCK-8 assay, and the IC_50_ was calculated by GraphPad Prism software data analysis. The results indicate that LSR overexpression induces cisplatin resistant to MDA-MB-231 cells, whereas LSR knockout sensitized MDA-MB-468 cells to cisplatin.
(TIF)

S3 Fig Localization of LSR in the nucleus to support its potential effects on gene transcription. Immunofluorescence staining of LSR in 231/C and 231/LSR cells was performed. Representative images of LSR (red) and nuclear staining with DAPI (blue) are shown.
(TIF)

S4 Fig Raw images from Western blot analysis. (PDF)

## References

[1] Yen FT, Masson M, Clossais-Besnard N, André P, Grosset JM, Bougueleret L, et al. Molecular cloning of a lipolysis-stimulated remnant receptor expressed in the liver. J Biol Chem. 1999;274(19):13390–8. doi: 10.1074/jbc.274.19.13390 10224102

[2] Yen FT, Roitel O, Bonnard L, Notet V, Pratte D, Stenger C, et al. Lipolysis stimulated lipoprotein receptor: a novel molecular link between hyperlipidemia, weight gain, and atherosclerosis in mice. J Biol Chem. 2008;283(37):25650–9. doi: 10.1074/jbc.M801027200 18644789

[3] Masuda S, Oda Y, Sasaki H, Ikenouchi J, Higashi T, Akashi M, et al. LSR defines cell corners for tricellular tight junction formation in epithelial cells. J Cell Sci. 2011;124(Pt 4):548–55. doi: 10.1242/jcs.072058 21245199

[4] Kohno T, Konno T, Kojima T. Role of tricellular tight junction protein lipolysis-stimulated lipoprotein receptor (LSR) in cancer cells. Int J Mol Sci. 2019;20(14). 10.3390/ijms20143555 PMC6679224 31330820

[5] Reaves DK, Hoadley KA, Fagan-Solis KD, Jima DD, Bereman M, Thorpe L, et al. Nuclear Localized LSR: A Novel Regulator of Breast Cancer Behavior and Tumorigenesis. Mol Cancer Res. 2017;15(2):165–78. doi: 10.1158/1541-7786.MCR-16-0085-T 27856957 PMC5290211

[6] Reaves DK, Fagan-Solis KD, Dunphy K, Oliver SD, Scott DW, Fleming JM. The role of lipolysis stimulated lipoprotein receptor in breast cancer and directing breast cancer cell behavior. PLoS One. 2014;9(3):e91747. doi: 10.1371/journal.pone.0091747 24637461 PMC3956714

[7] Leth-Larsen R, Terp MG, Christensen AG, Elias D, Kühlwein T, Jensen ON, et al. Functional heterogeneity within the CD44 high human breast cancer stem cell-like compartment reveals a gene signature predictive of distant metastasis. Mol Med. 2012;18(1):1109–21. doi: 10.2119/molmed.2012.00091 22692575 PMC3474436

[8] Wang J-Q, Yang Y, Cai C-Y, Teng Q-X, Cui Q, Lin J, et al. Multidrug resistance proteins (MRPs): Structure, function and the overcoming of cancer multidrug resistance. Drug Resist Updat. 2021;54:100743. doi: 10.1016/j.drup.2021.100743 33513557

[9] Nandi SK, Roychowdhury T, Chattopadhyay S, Basu S, Chatterjee K, Choudhury P, et al. Deregulation of the CD44-NANOG-MDR1 associated chemoresistance pathways of breast cancer stem cells potentiates the anti-cancer effect of Kaempferol in synergism with Verapamil. Toxicol Appl Pharmacol. 2022;437:115887. doi: 10.1016/j.taap.2022.115887 35063459

[10] Shi Y, Ma Z, Cheng Q, Wu Y, Parris AB, Kong L, et al. FGFR1 overexpression renders breast cancer cells resistant to metformin through activation of IRS1/ERK signaling. Biochim Biophys Acta Mol Cell Res. 2021;1868(1):118877. doi: 10.1016/j.bbamcr.2020.118877 33007330

[11] Cheng Q, Ma Z, Shi Y, Parris AB, Kong L, Yang X. FGFR1 Overexpression Induces Cancer Cell Stemness and Enhanced Akt/Erk-ER Signaling to Promote Palbociclib Resistance in Luminal A Breast Cancer Cells. Cells. 2021;10(11):3008. doi: 10.3390/cells10113008 34831231 PMC8616148

[12] Zhao M, Howard EW, Guo Z, Parris AB, Yang X. p53 pathway determines the cellular response to alcohol-induced DNA damage in MCF-7 breast cancer cells. PLoS One. 2017;12(4):e0175121. doi: 10.1371/journal.pone.0175121 28369097 PMC5378409

[13] Mazumder S, Plesca D, Almasan A. Caspase-3 activation is a critical determinant of genotoxic stress-induced apoptosis. Apoptosis and Cancer: Methods and Protocols. 2008. 13–21. 10.1007/978-1-59745-339-4_2 18175808

[14] Carreira ASA, Ravera S, Zucal C, Thongon N, Caffa I, Astigiano C, et al. Mitochondrial rewiring drives metabolic adaptation to NAD(H) shortage in triple negative breast cancer cells. Neoplasia. 2023;41:100903. doi: 10.1016/j.neo.2023.100903 37148658 PMC10192916

[15] Wang C-W, Huang C-C, Chou P-H, Chang Y-P, Wei S, Guengerich FP, et al. 7-ketocholesterol and 27-hydroxycholesterol decreased doxorubicin sensitivity in breast cancer cells: estrogenic activity and mTOR pathway. Oncotarget. 2017;8(39):66033–50. doi: 10.18632/oncotarget.19789 29029490 PMC5630390

[16] Kanda M, Serada S, Hiramatsu K, Funauchi M, Obata K, Nakagawa S, et al. Lipolysis-stimulated lipoprotein receptor-targeted antibody-drug conjugate demonstrates potent antitumor activity against epithelial ovarian cancer. Neoplasia. 2023;35:100853. doi: 10.1016/j.neo.2022.100853 36413881 PMC9679668

[17] Devarajan E, Sahin AA, Chen JS, Krishnamurthy RR, Aggarwal N, Brun A-M, et al. Down-regulation of caspase 3 in breast cancer: a possible mechanism for chemoresistance. Oncogene. 2002;21(57):8843–51. doi: 10.1038/sj.onc.1206044 12483536

[18] Kimura Y, Morita S, Matsuo M, Ueda K. Mechanism of multidrug recognition by MDR1/ABCB1. Cancer Sci. 2007;98(9):1303–10. doi: 10.1111/j.1349-7006.2007.00538.x 17608770 PMC11159003

