# Supplementary figures and images for: LSR overexpression induces chemoresistance in triple negative breast cancer cells through MDR1 upregulation and apoptosis attenuation
Source: PLoS One. 2025 Nov 3;20(11):e0336124. doi: 10.1371/journal.pone.0336124 (PMC12582462; doi:10.1371/journal.pone.0336124)

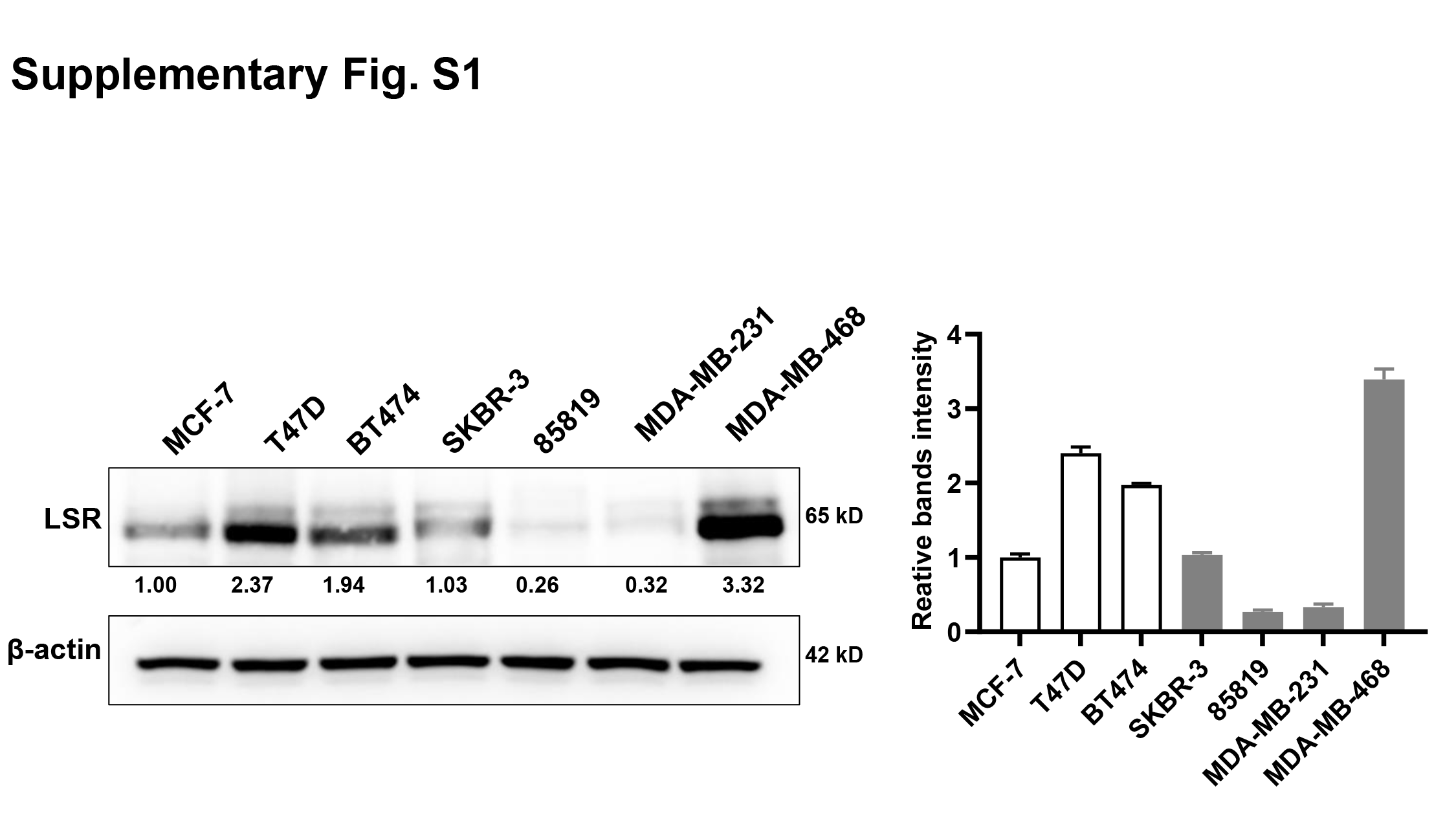

Supplement: S1 Fig — Protein lysates of indicated cell lines were prepared. Protein levels of LSR were detected with Western blotting and the relative LSR intensity was quantified. (TIF) [file pone.0336124.s001.tif]

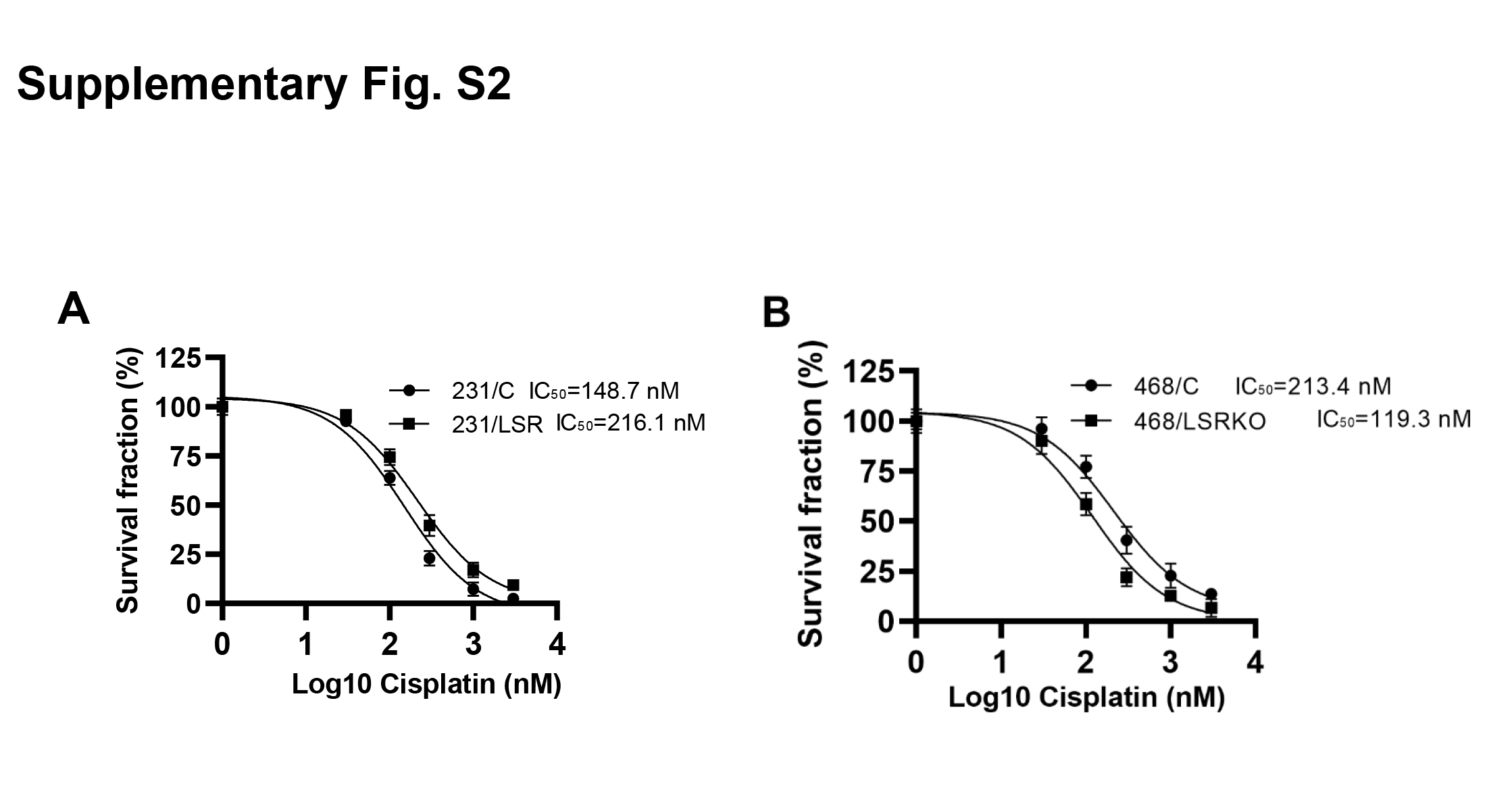

Supplement: S2 Fig — A & B) LSR overexpressing MDA-MB-231 cells (231/LSR), LSR knockout MDA-MB-468 cells (468/LSRKO) and corresponding control cells were seeded into 96-well plates at a density of 1000 cells/well. After 24 h of incubation, the cells were treated with different concentrations of cisplatin (0, 0.03, 0.1, 0.3, 1, 3 μM) for four days. The cell survival was evaluated by CCK-8 assay, and the IC50 was calculated by GraphPad Prism software data analysis. The results indicate that LSR overexpression induces cisplatin resistant to MDA-MB-231 cells, whereas LSR knockout sensitized MDA-MB-468 cells to cisplatin. (TIF) [file pone.0336124.s002.tif]

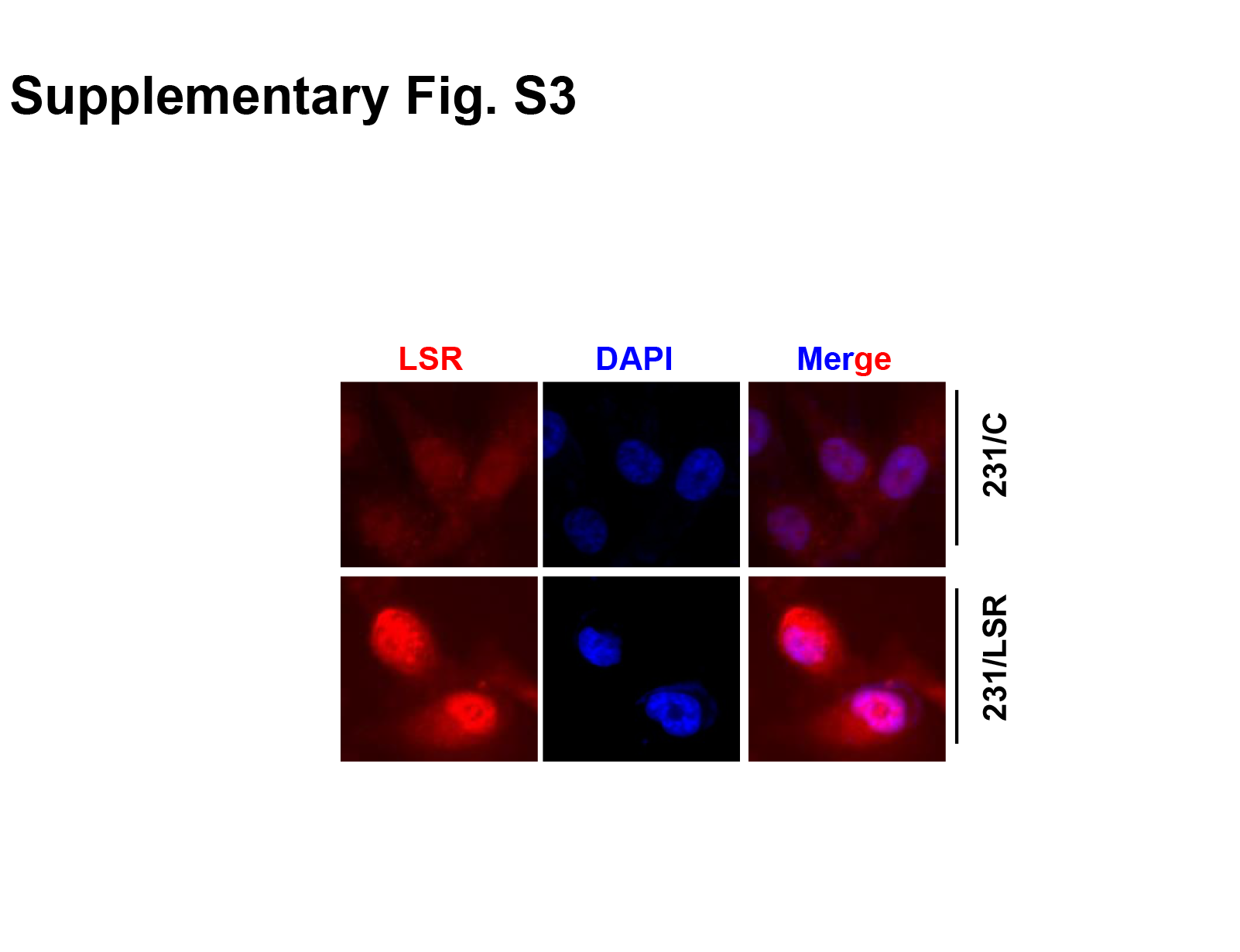

Supplement: S3 Fig — Immunofluorescence staining of LSR in 231/C and 231/LSR cells was performed. Representative images of LSR (red) and nuclear staining with DAPI (blue) are shown. (TIF) [file pone.0336124.s003.tif]
