# Supplementary material for: LSR overexpression induces chemoresistance in triple negative breast cancer cells through MDR1 upregulation and apoptosis attenuation
Source: PLoS One. 2025 Nov 3;20(11):e0336124. doi: 10.1371/journal.pone.0336124 (PMC12582462; doi:10.1371/journal.pone.0336124)

Fig1A-LSR

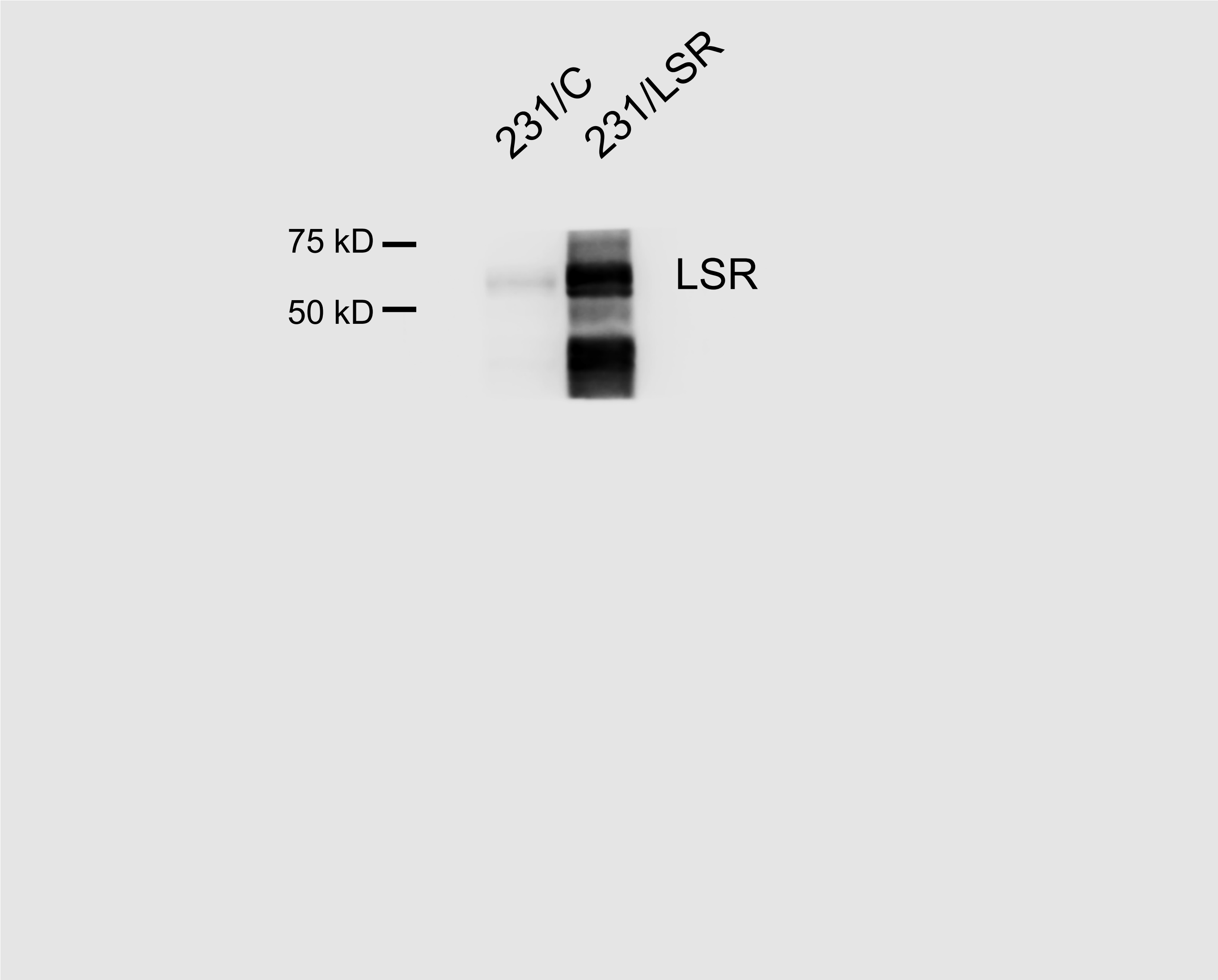

Fig1A-β-Actin

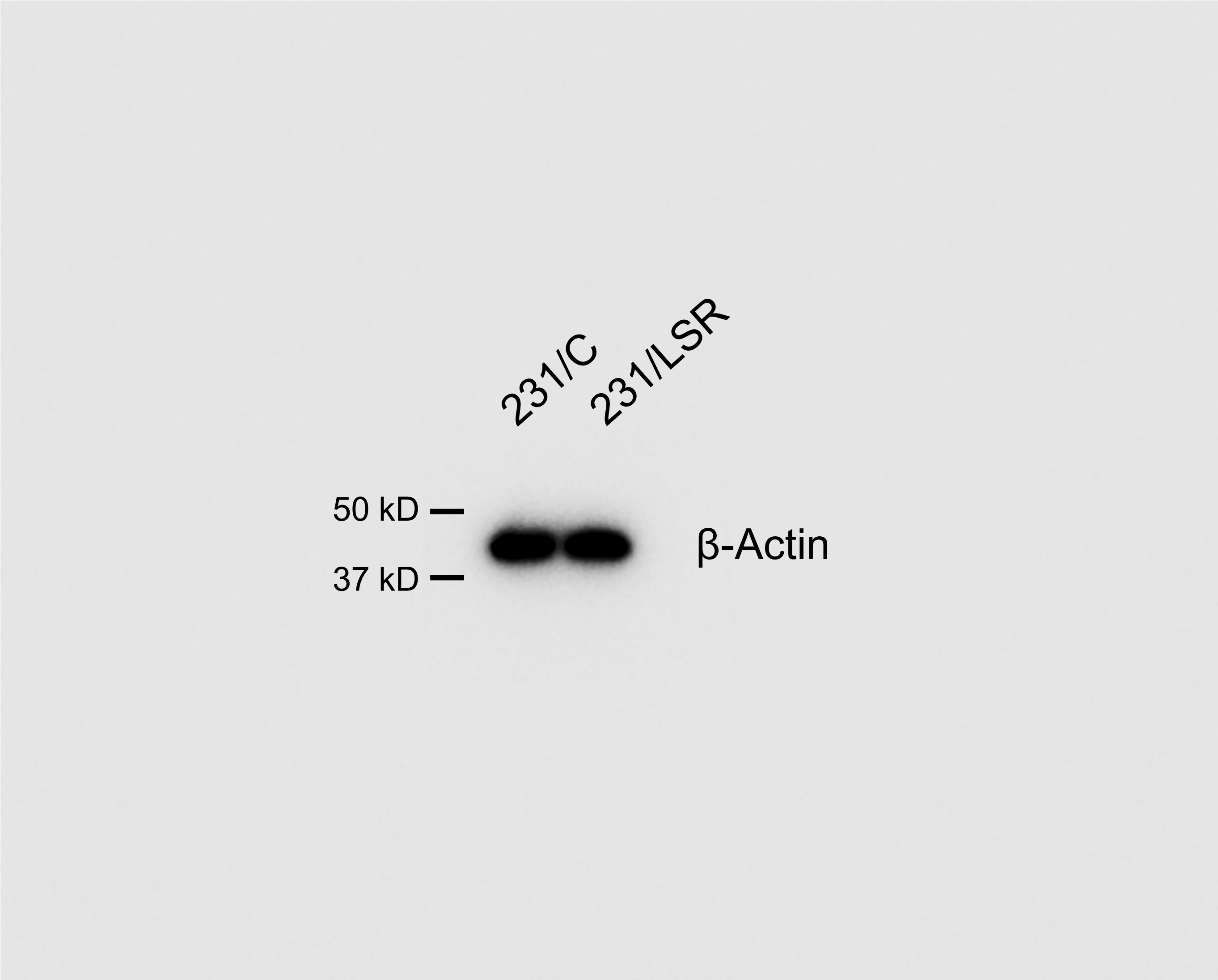

Fig2C-c-PARP

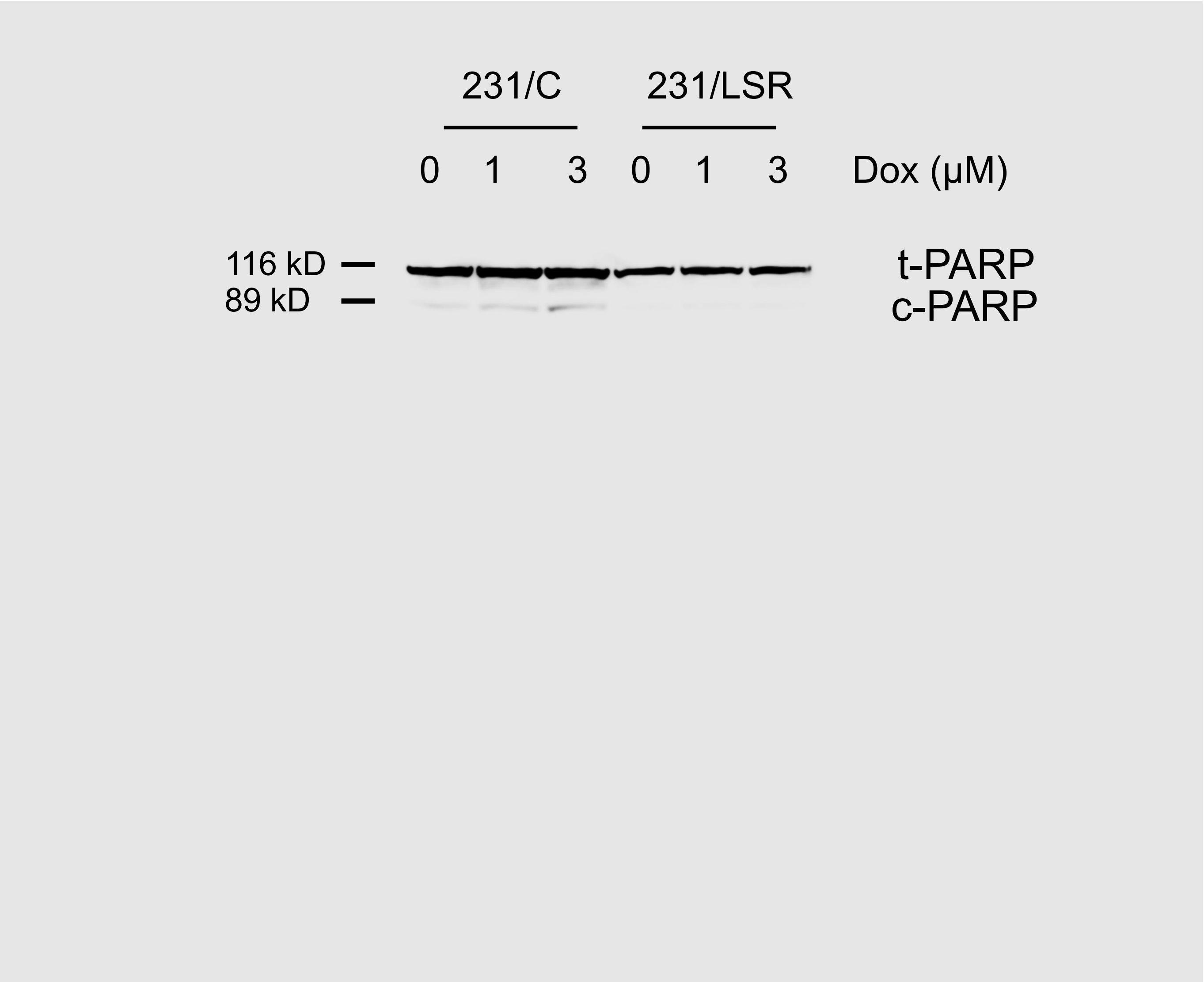

Fig2C-Casp3

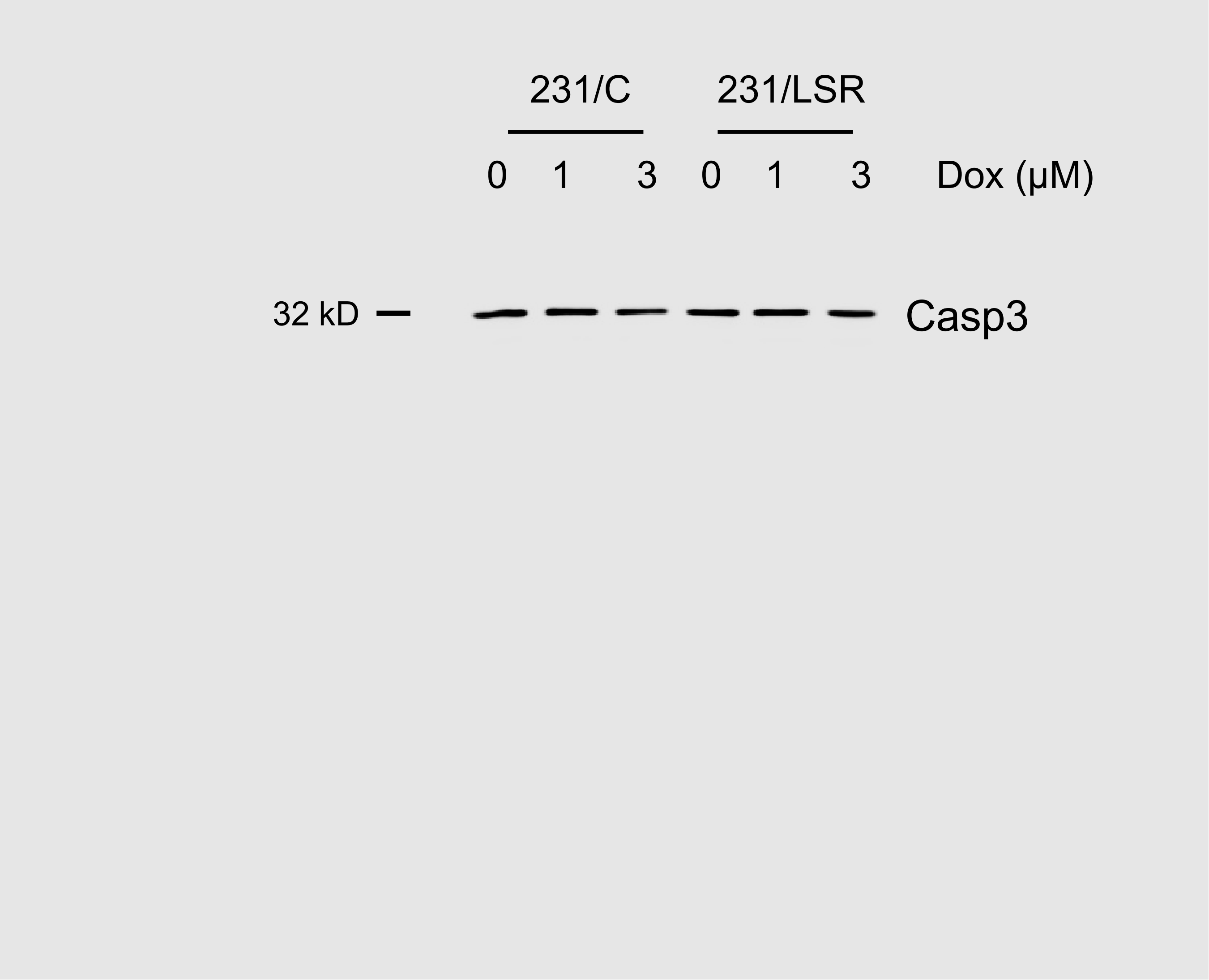

Fig2C-c-Casp3

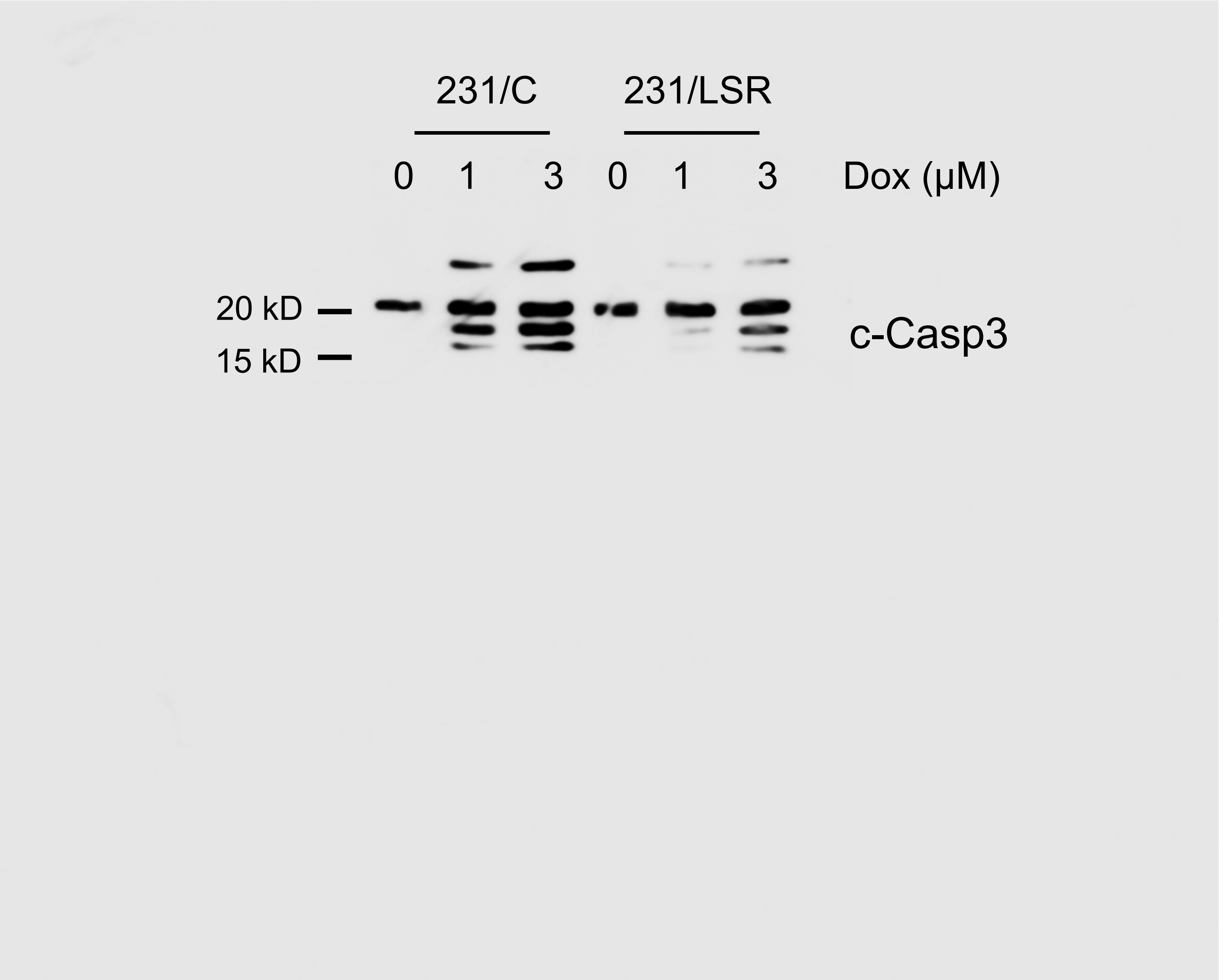

Fig2C-β-Actin

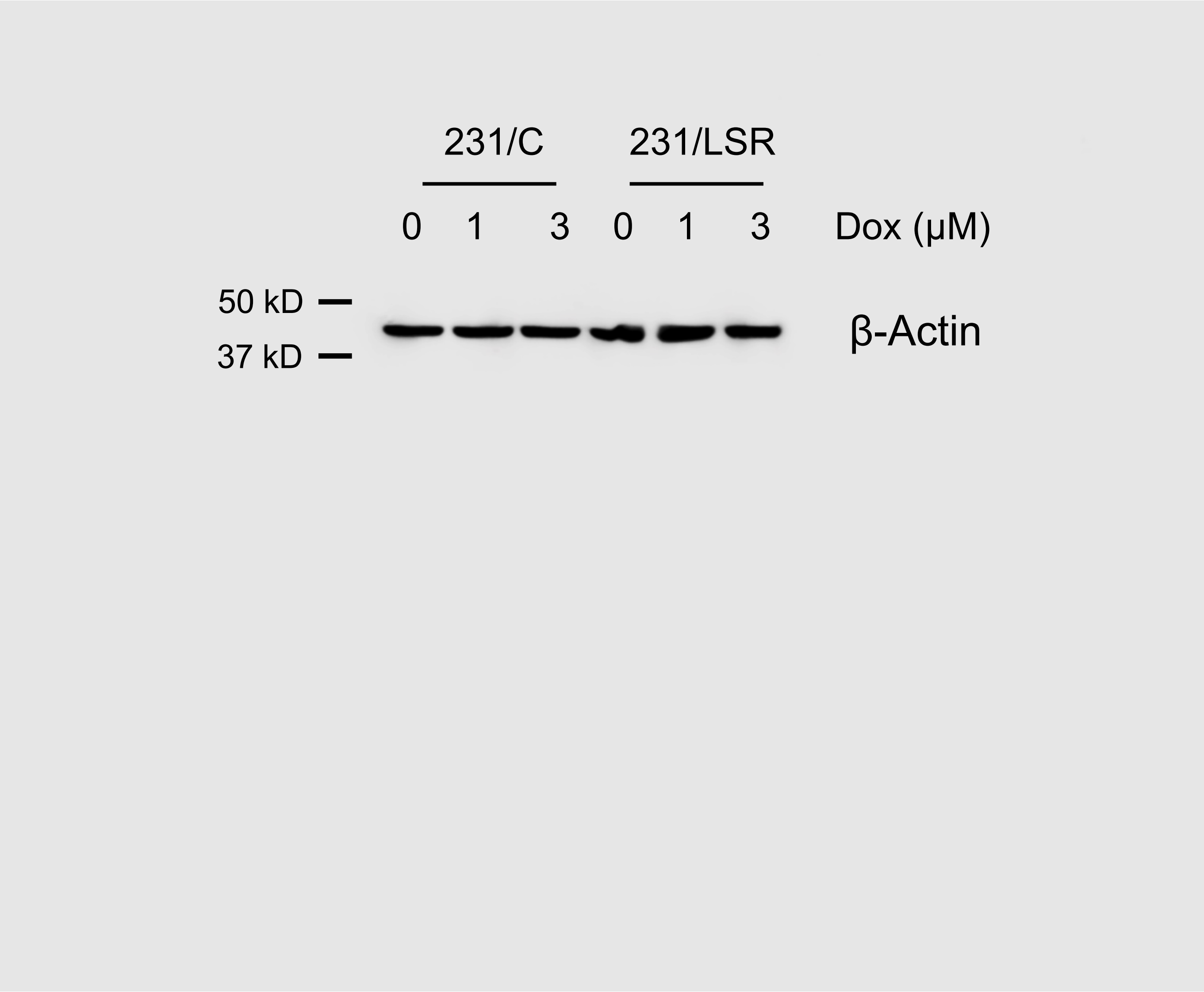

Fig3B-MDR1

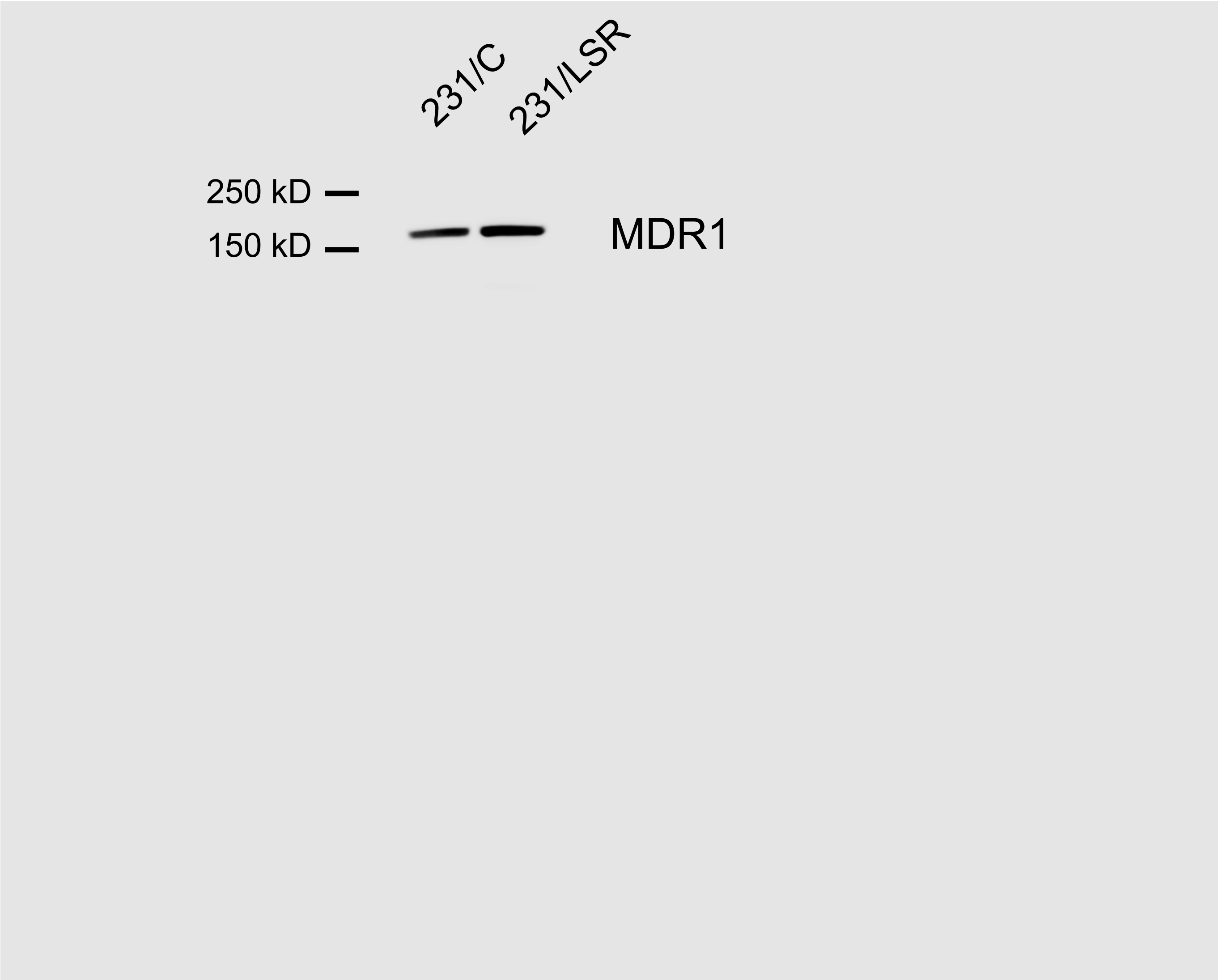

Fig3B-β-Actin

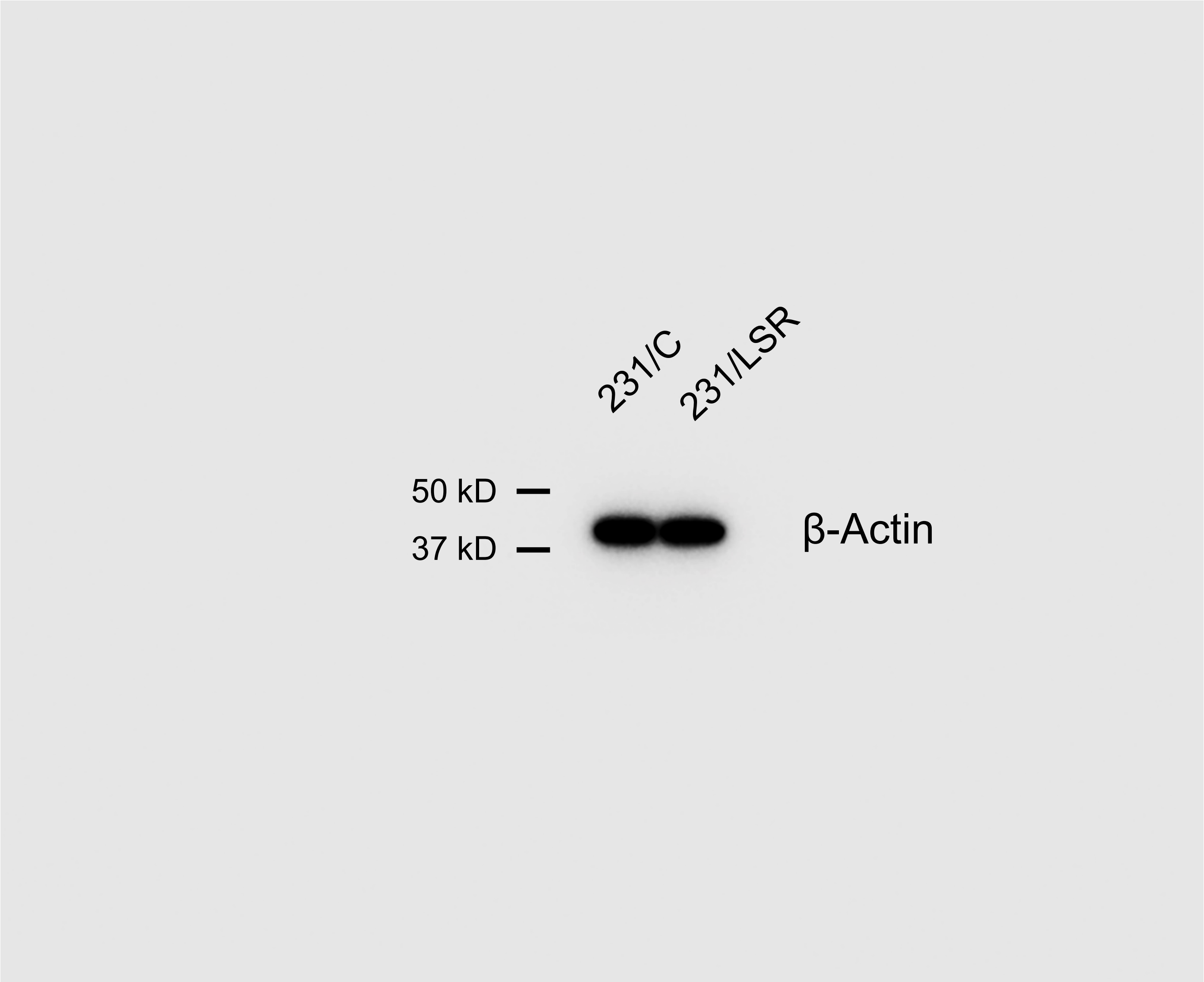

Fig3G-c-PARP

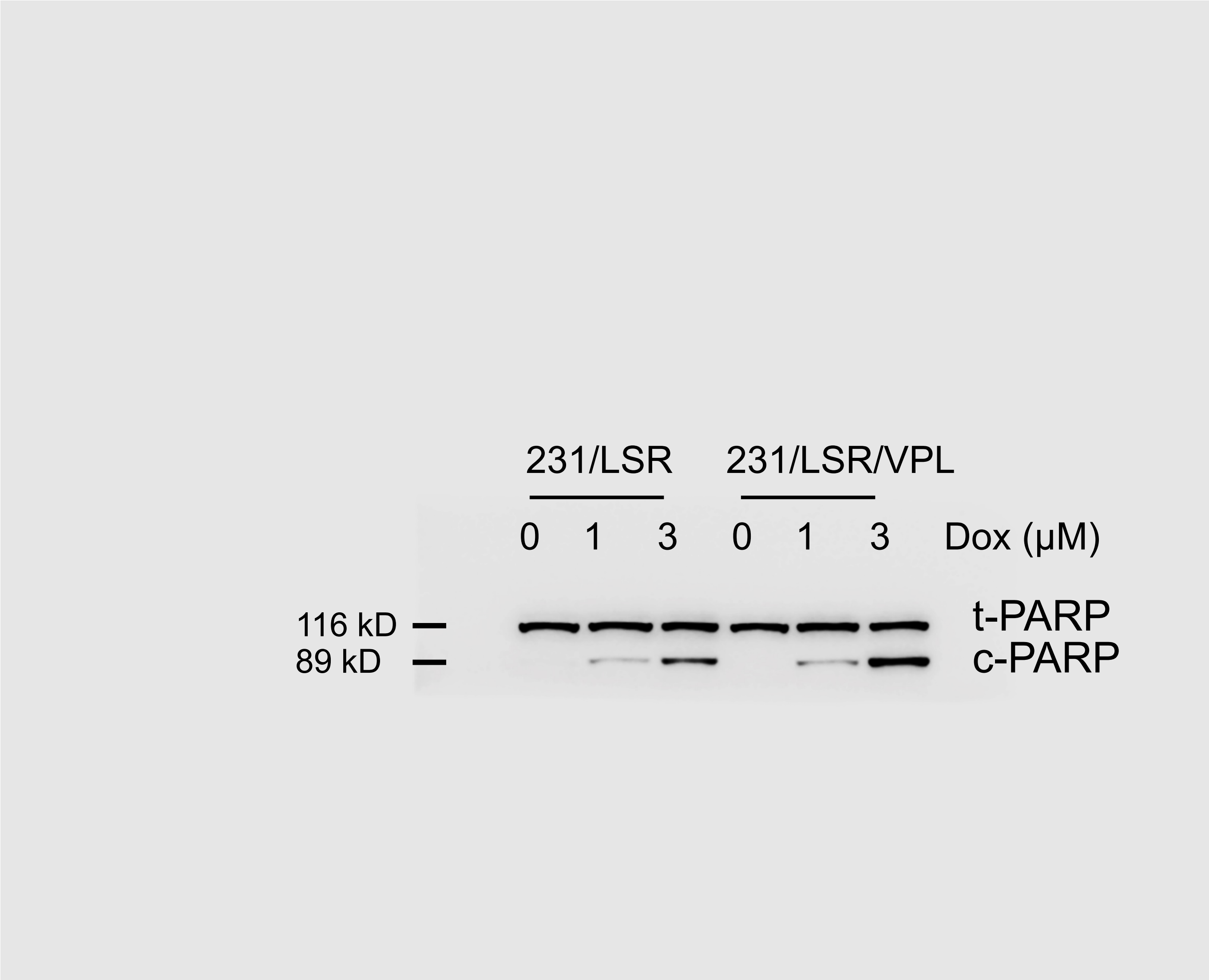

Fig3G-Casp3

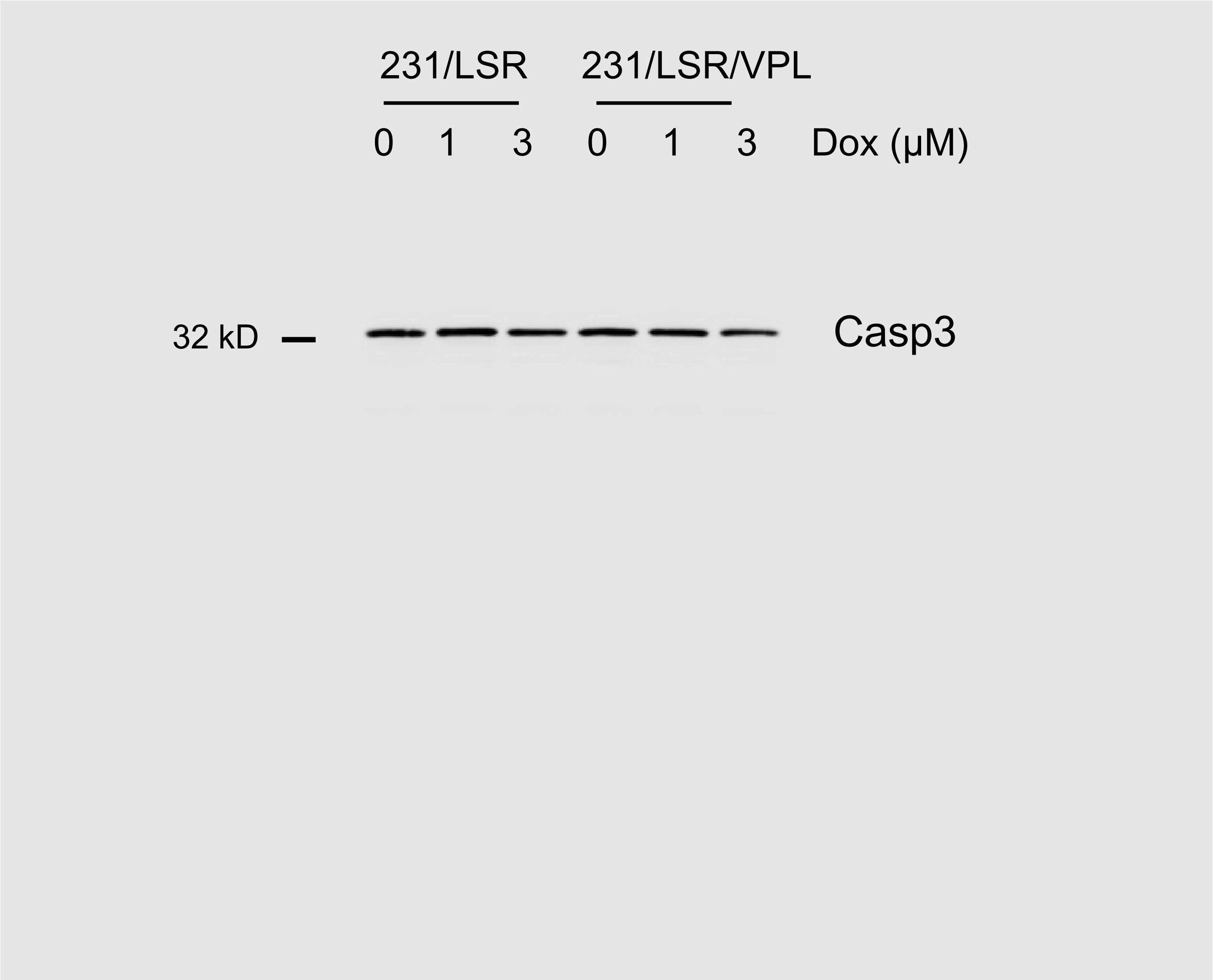

Fig3G-c-Casp3

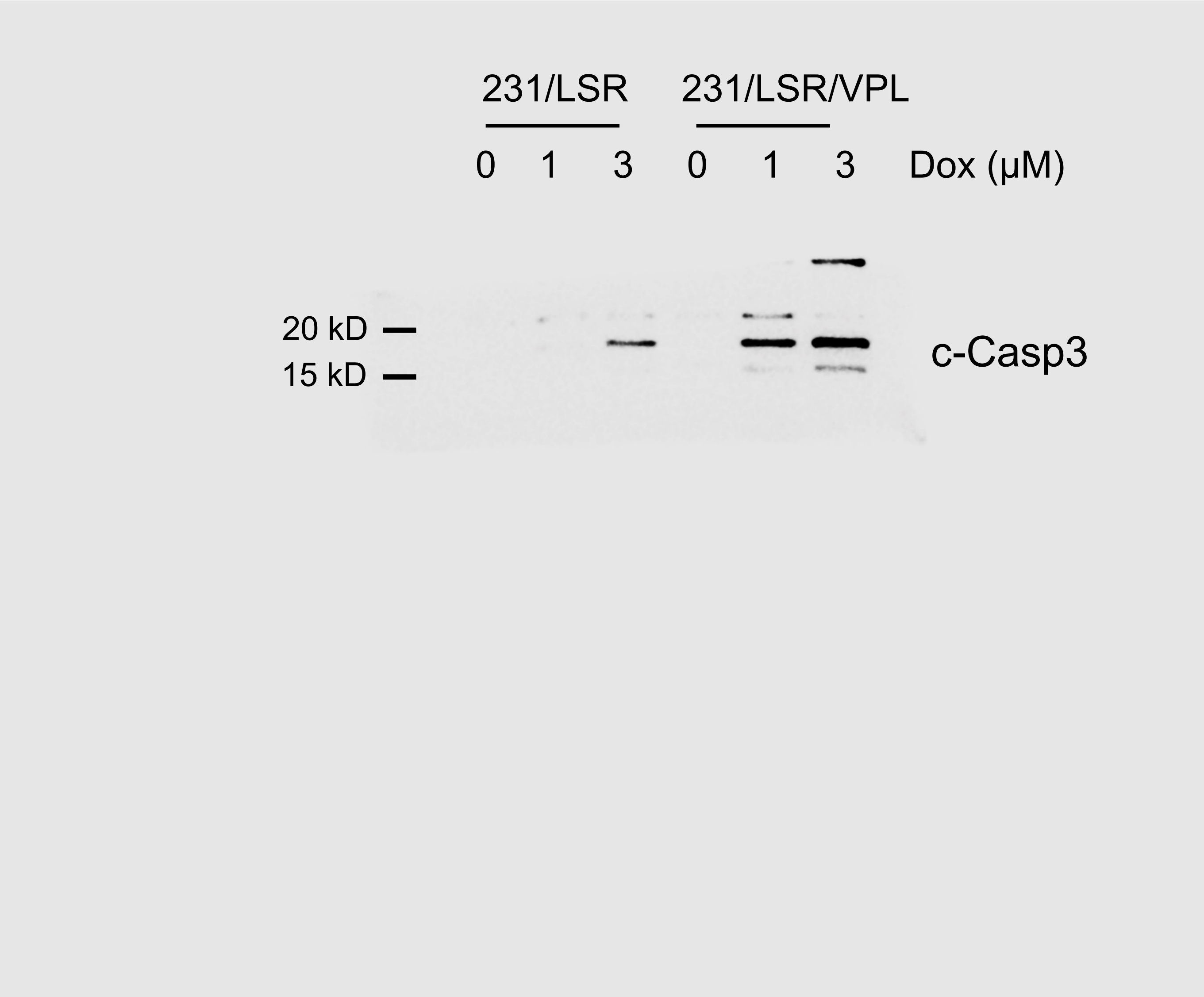

Fig3G-β-Actin

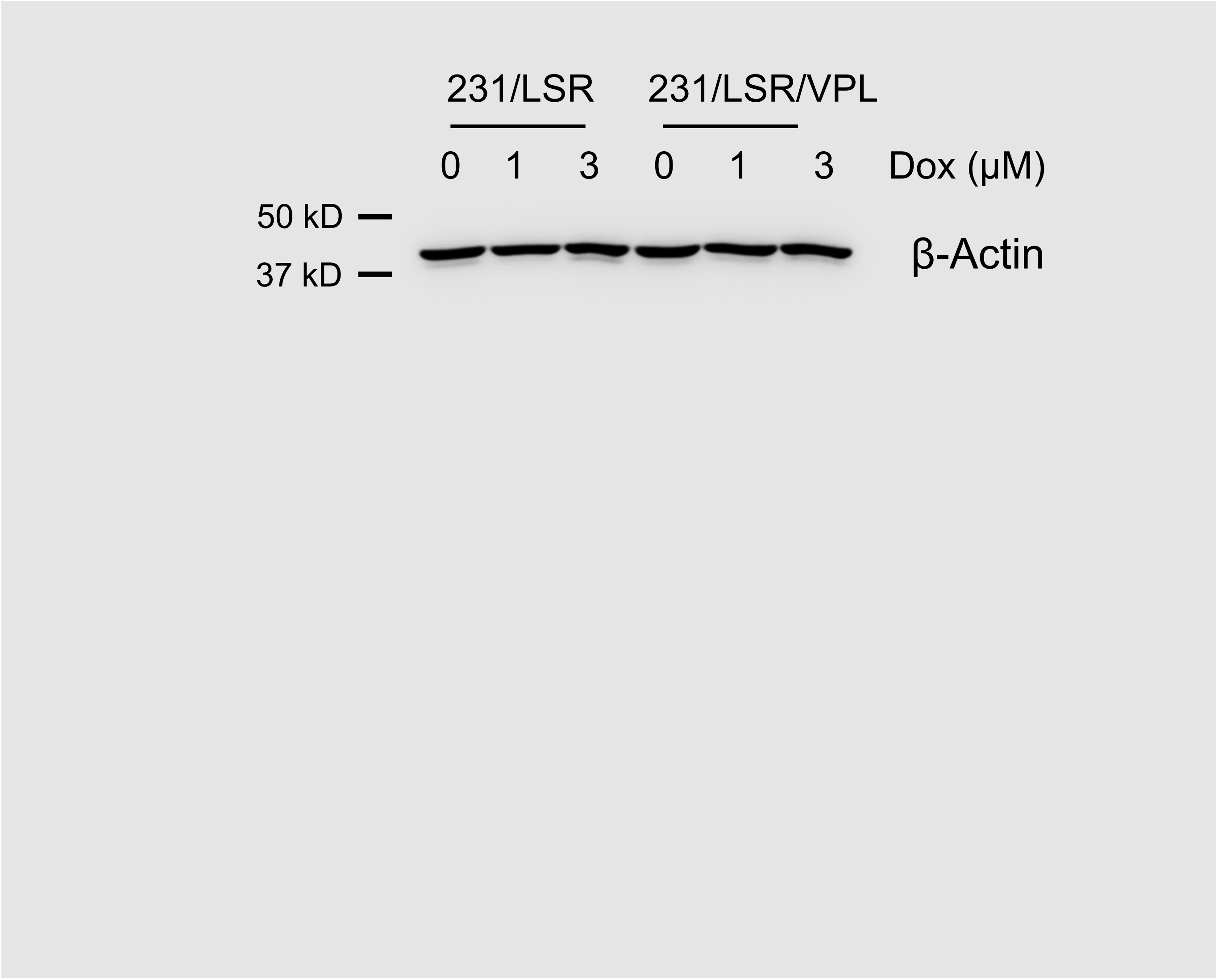

Fig.3H--MDR1

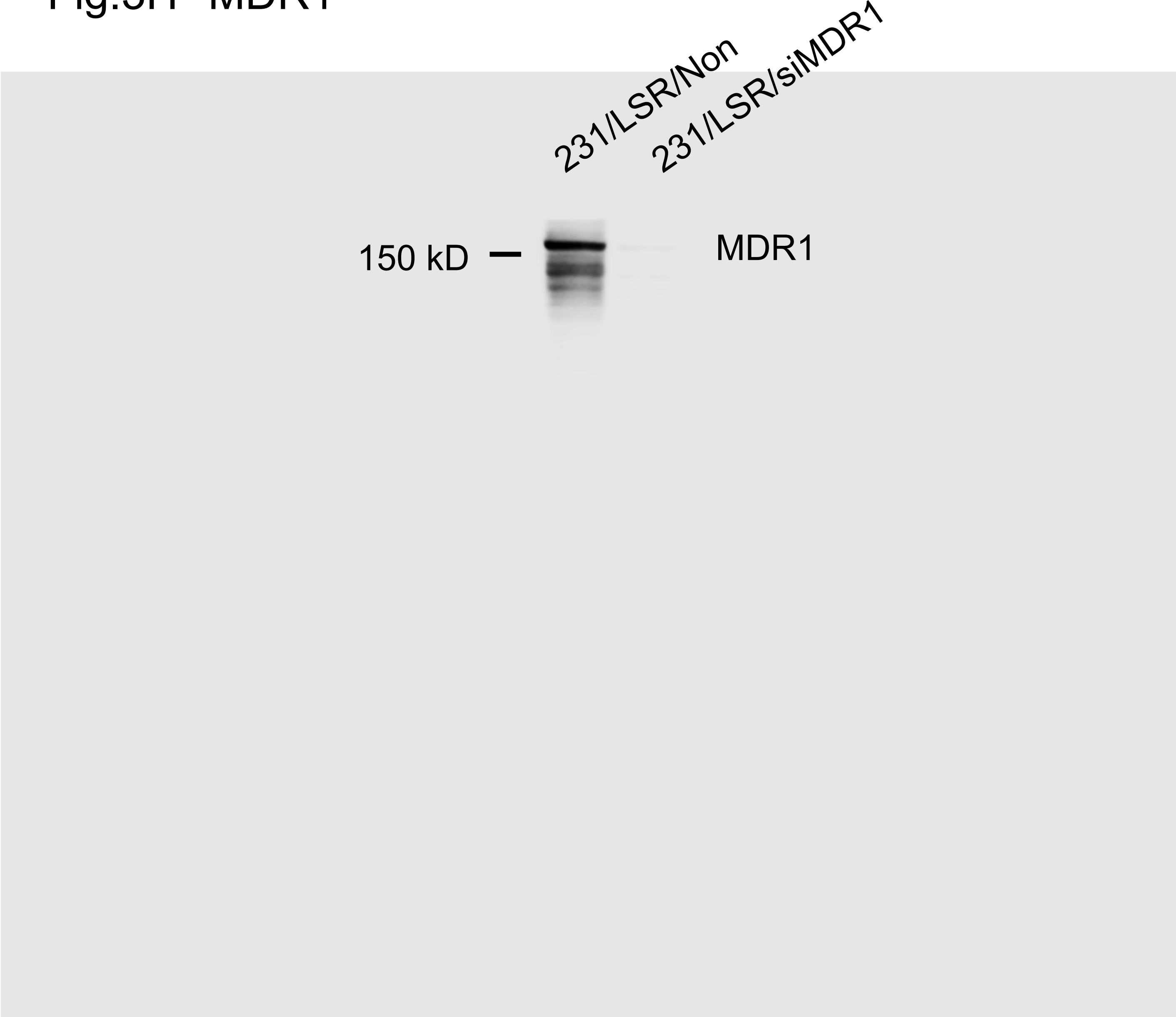

Fig.3H--β-Actin

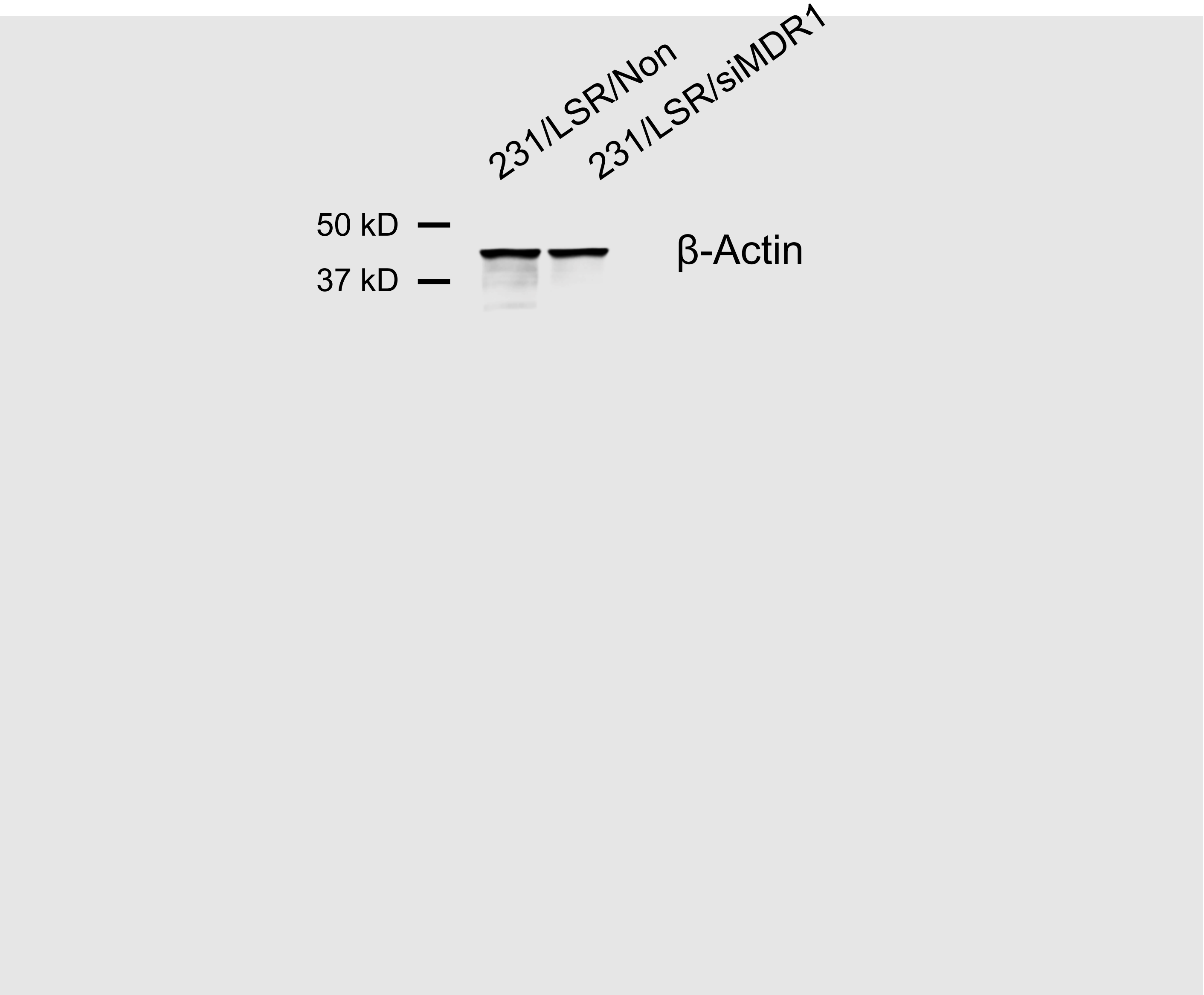

Fig.3J--PARP

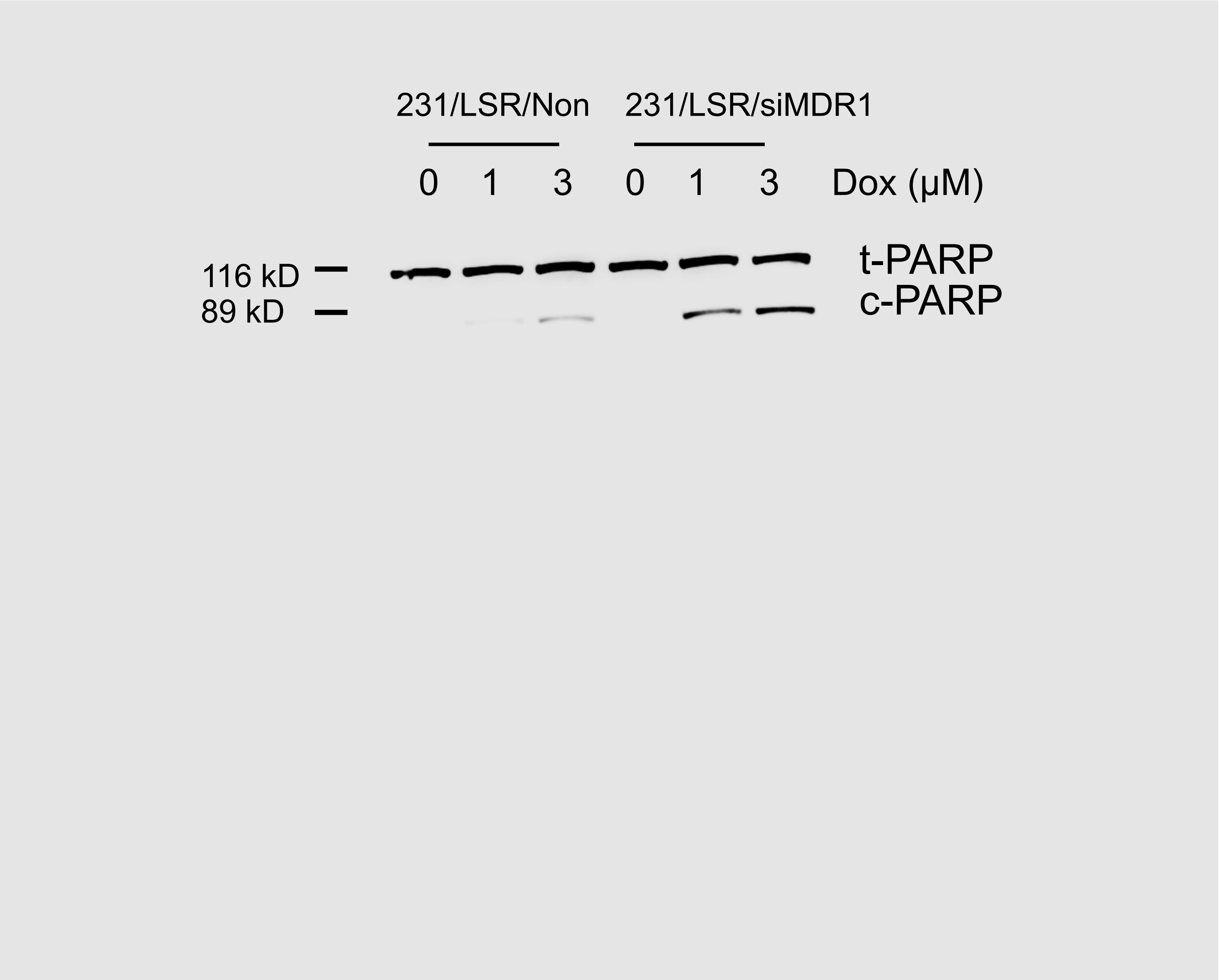
231/LSR/Non
231/LSR/siMDR1
0 1 3 0 1 3 Dox (μM)
116 kD
89 kD
t-PARP
c-PARP

Fig.3J--Casp3

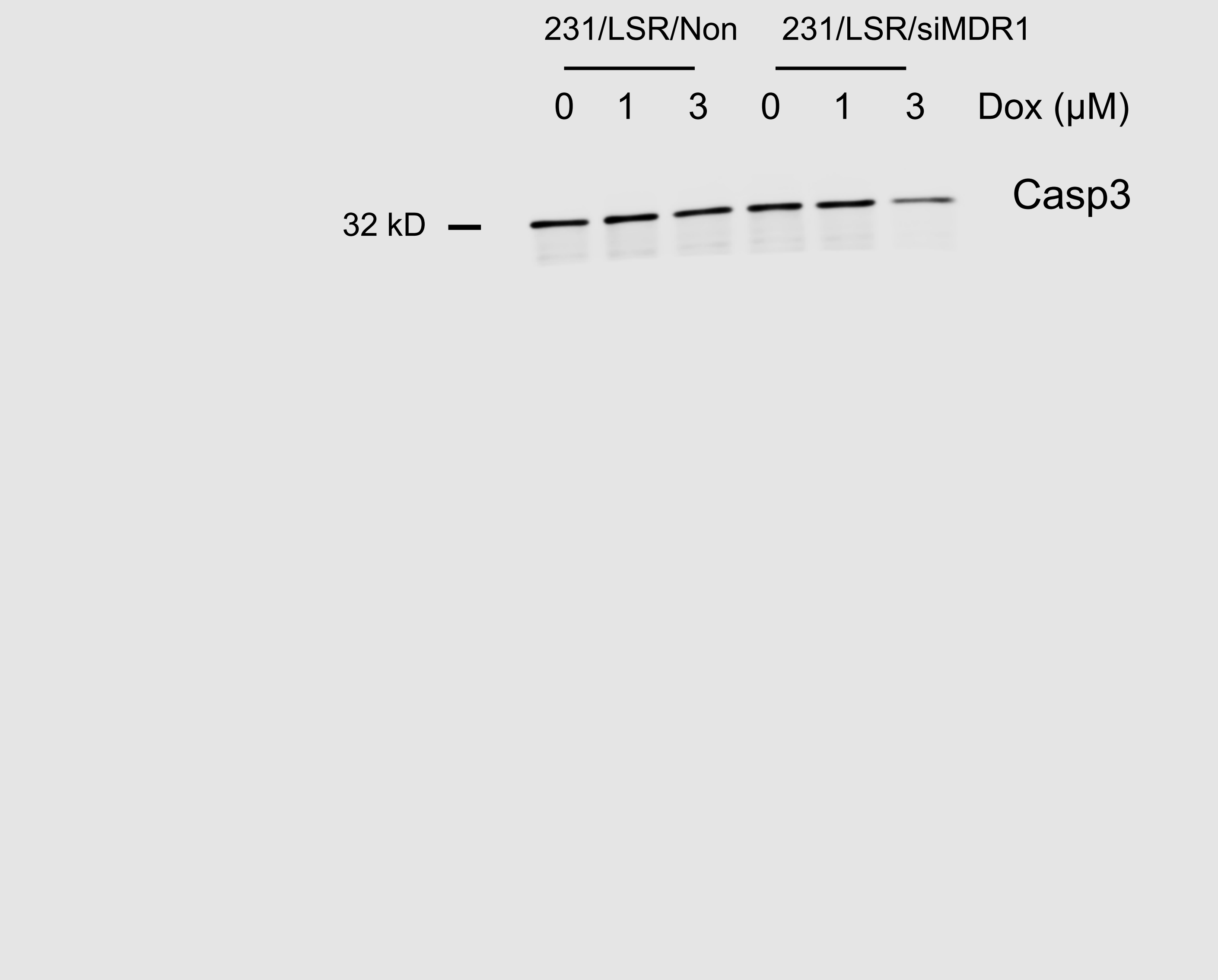

Fig.3J--c-Casp3

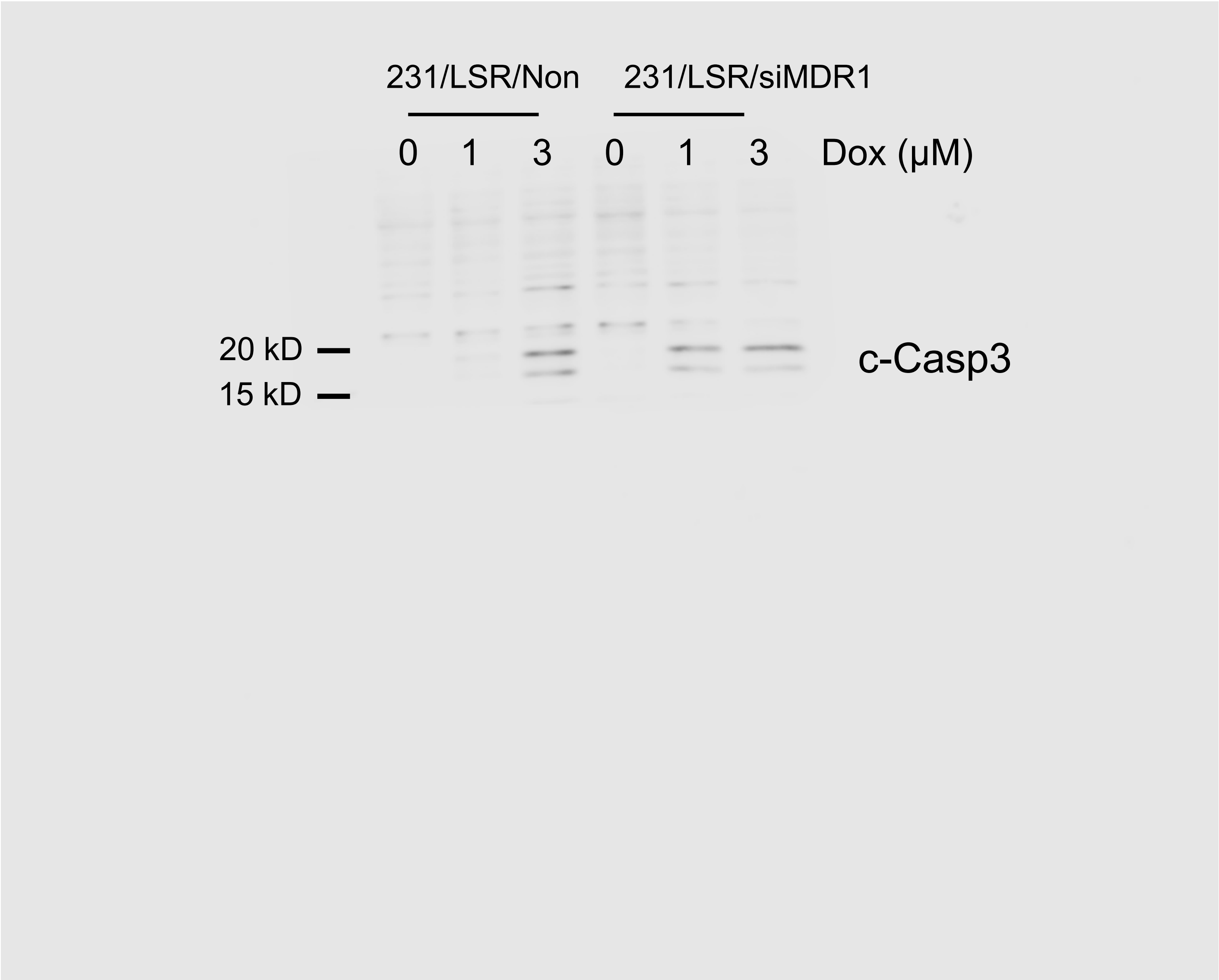

Fig.3J--β-Actin

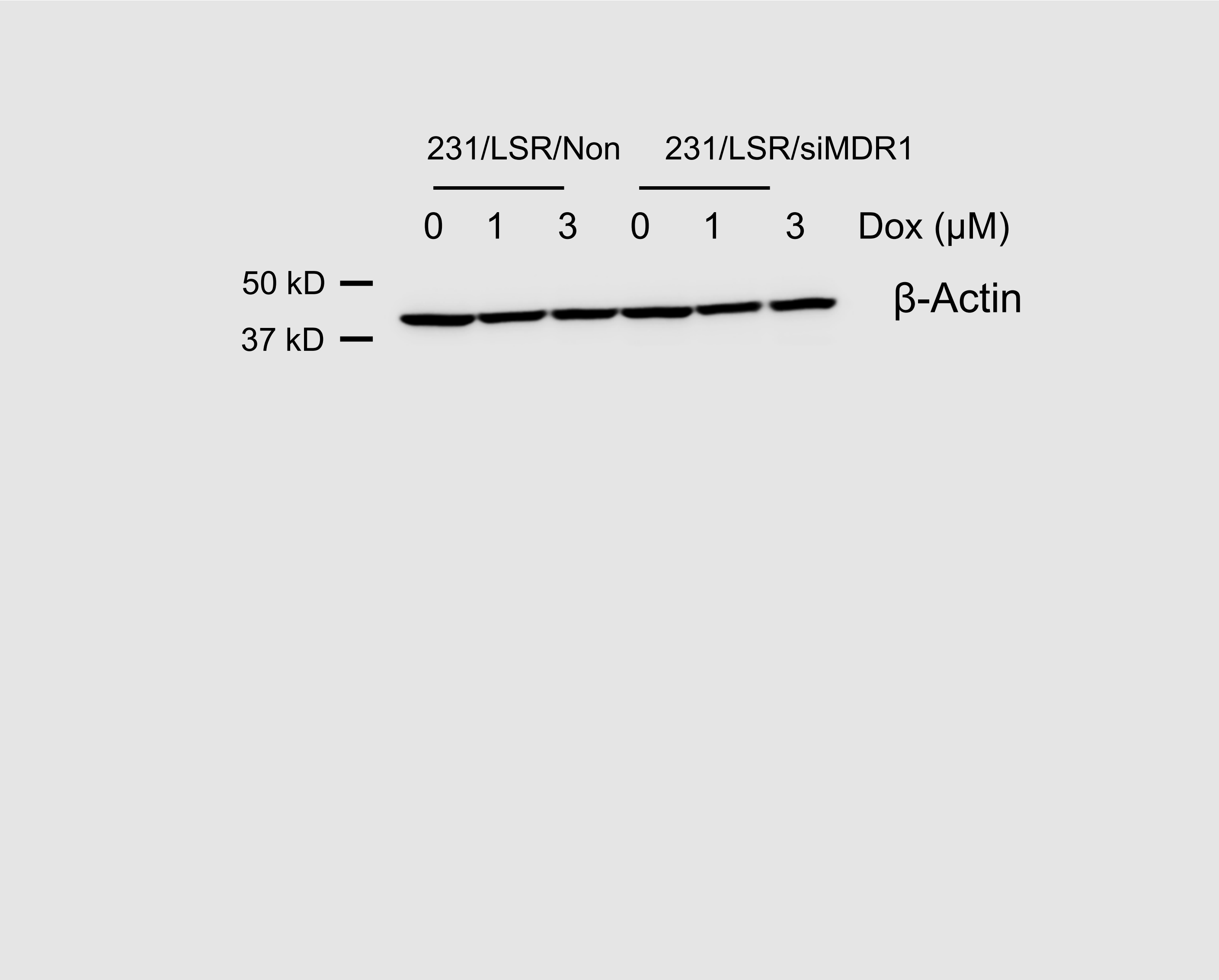

Fig4A-LSR

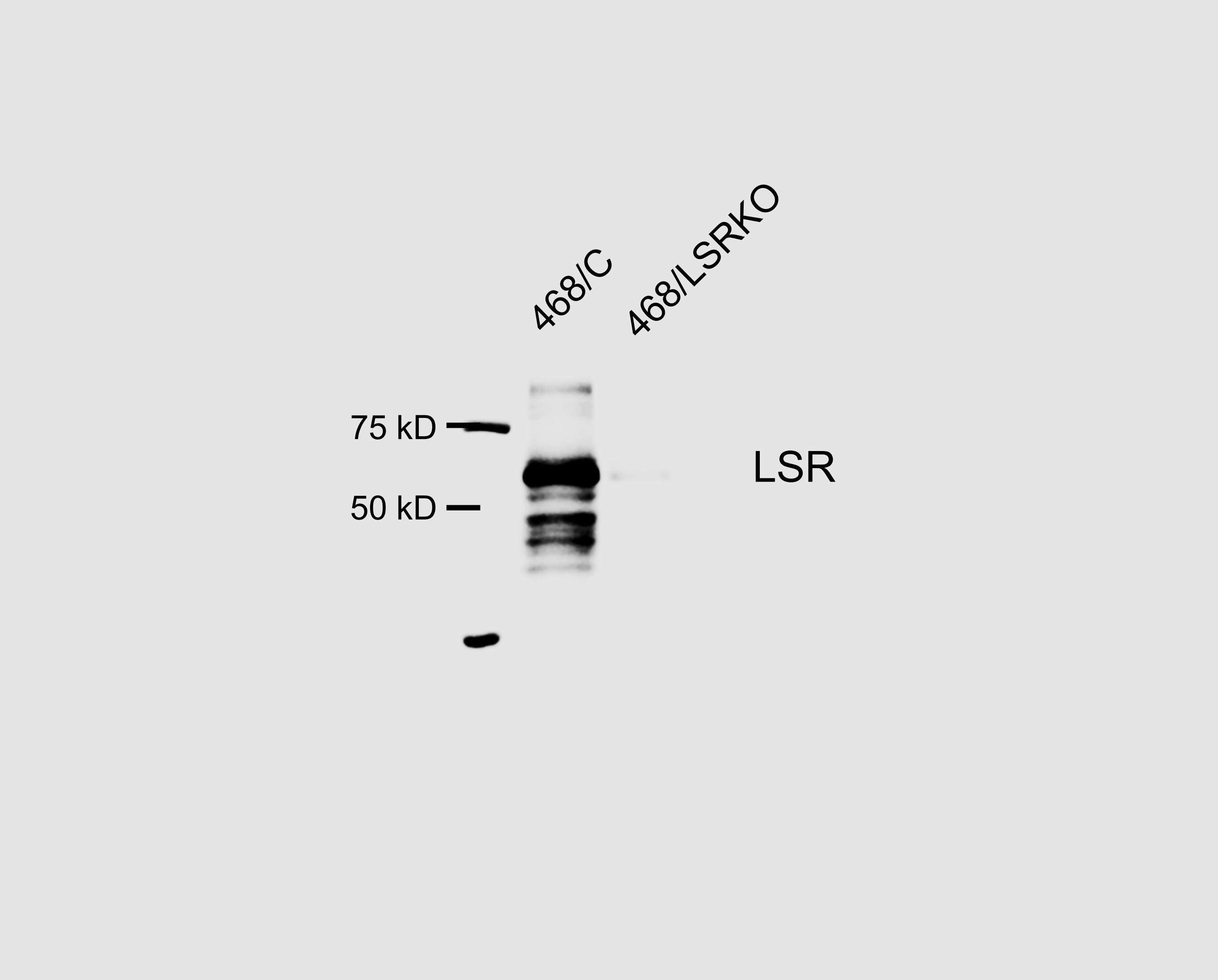

Fig4A-β-Actin

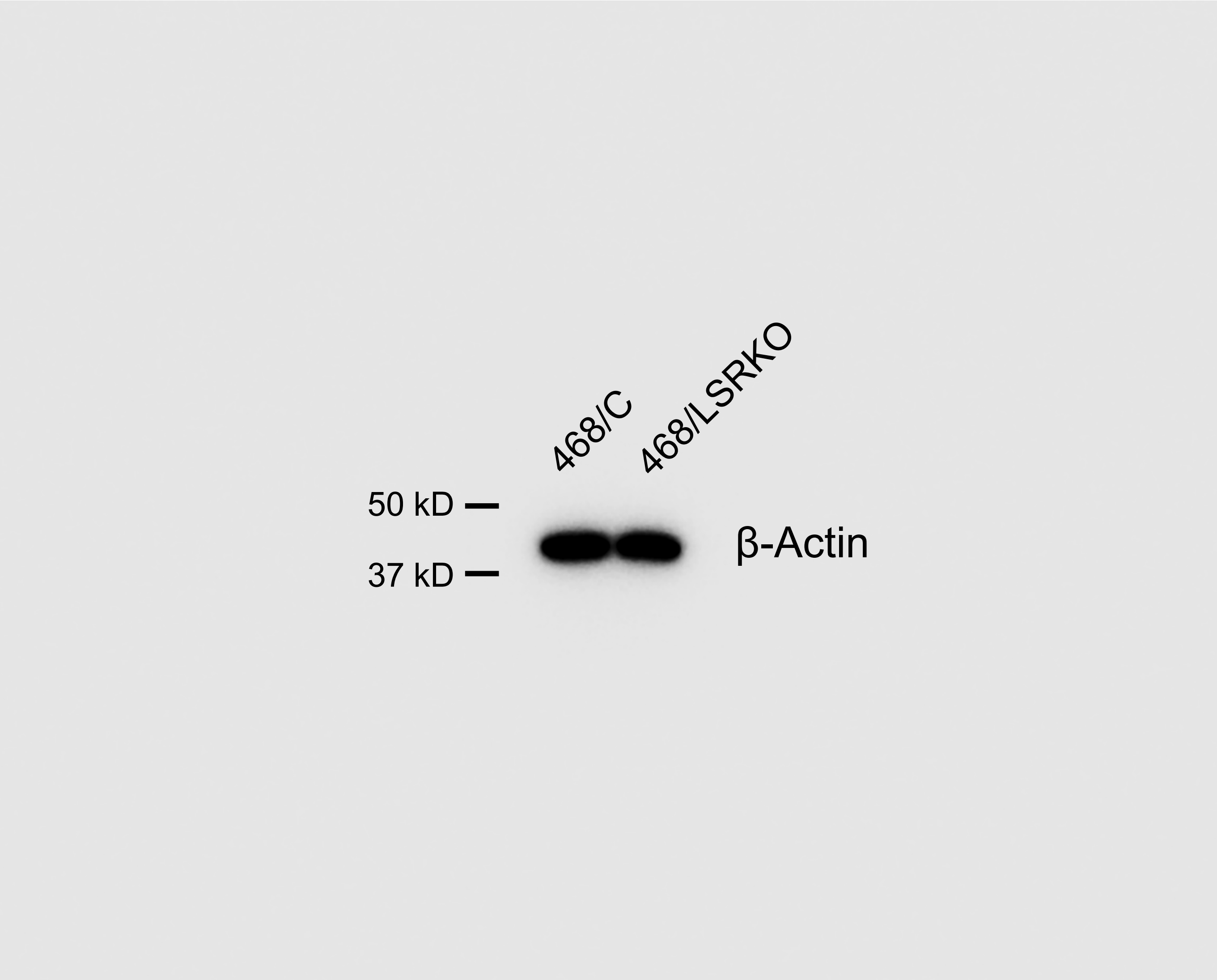

Fig4B-MDR1

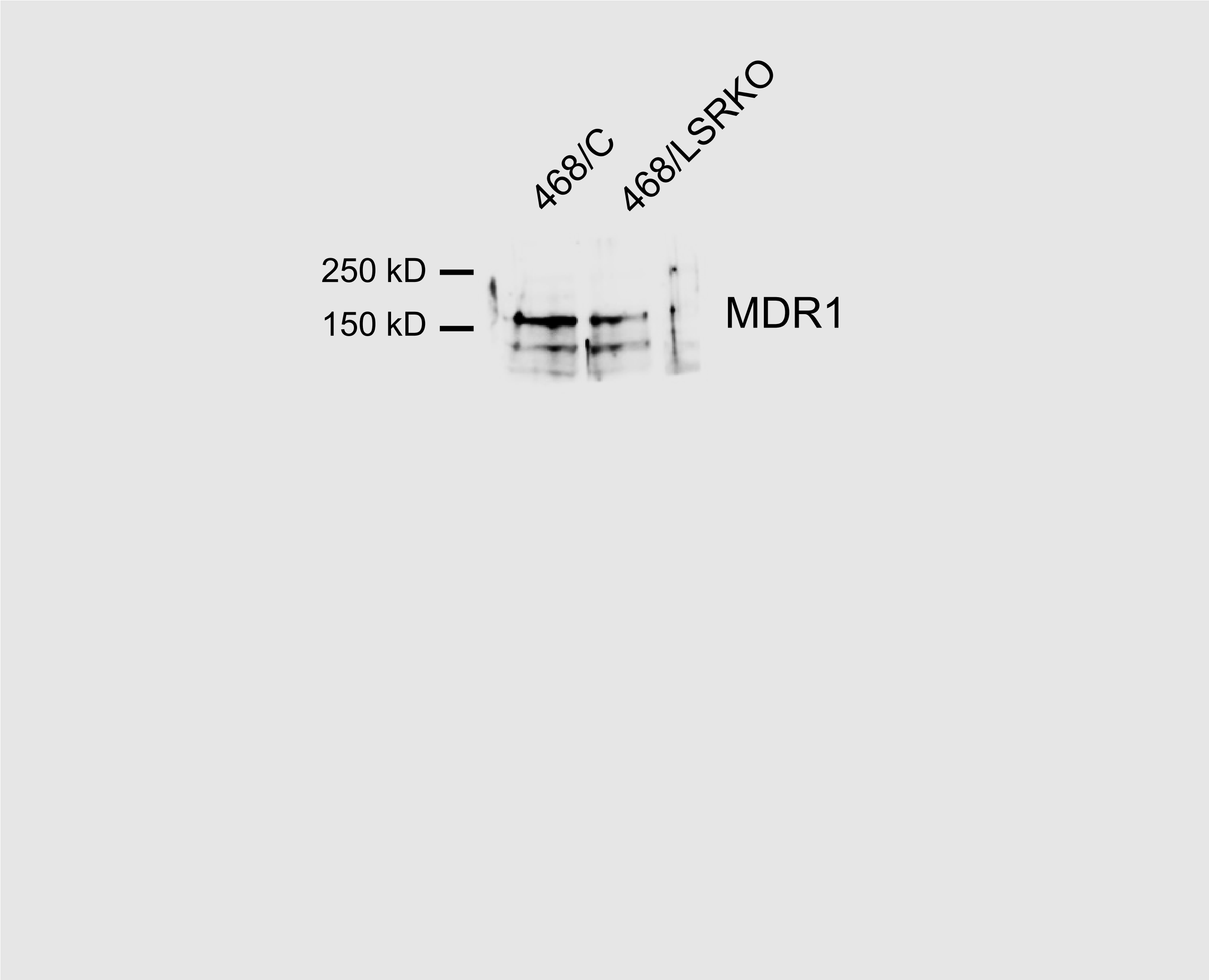

Fig4B-β-Actin

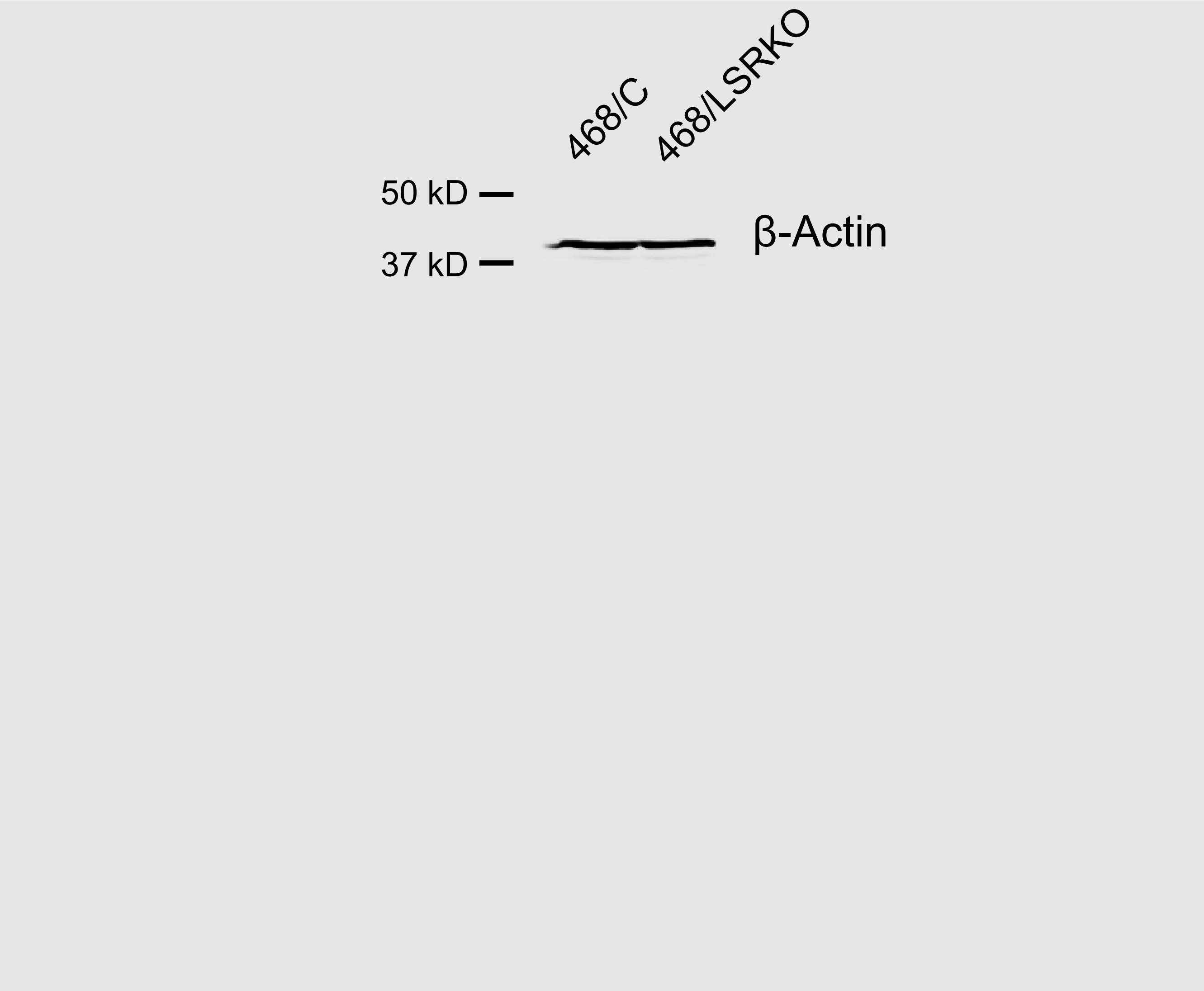

Fig4I-c-PARP

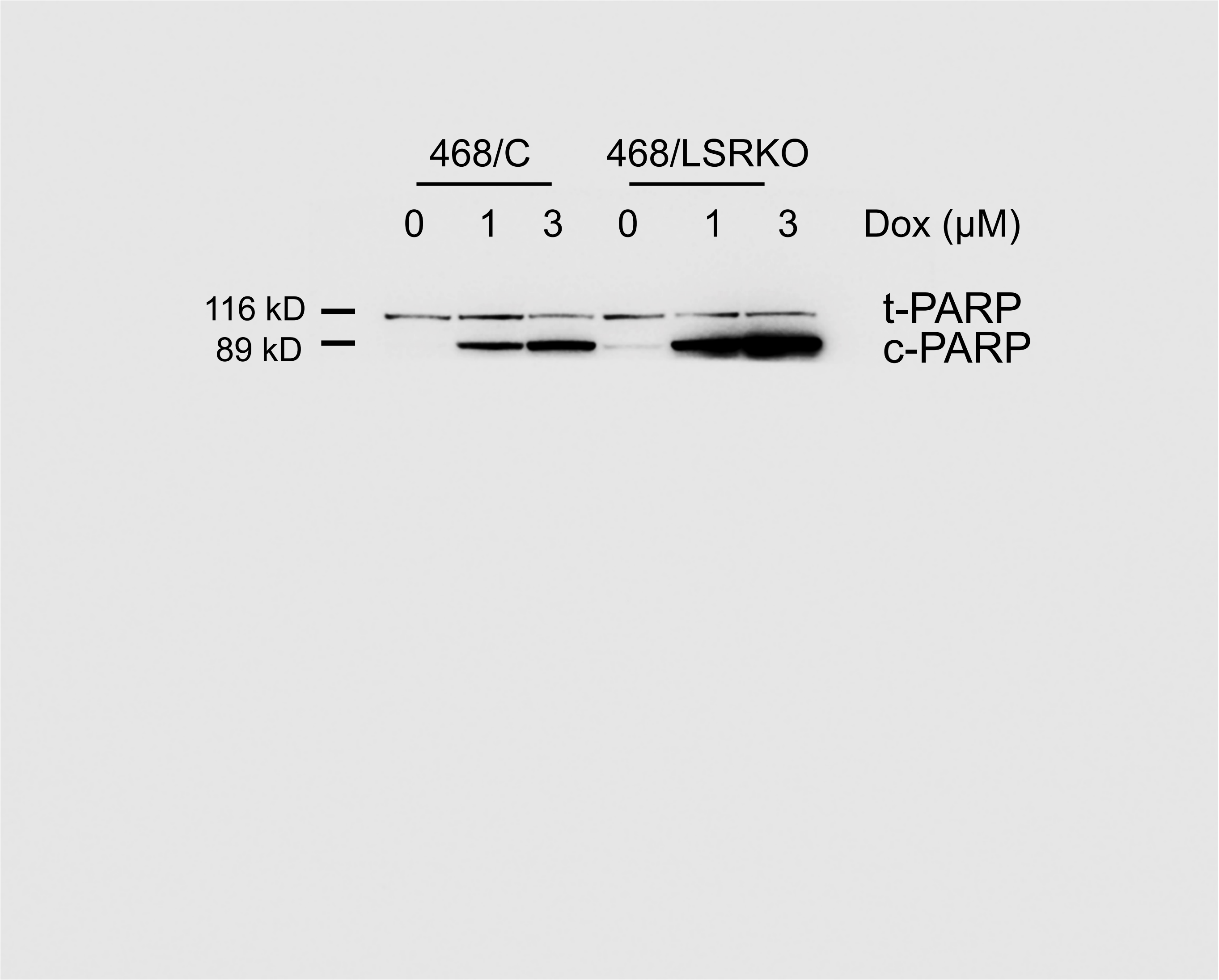

Fig4I-Casp3

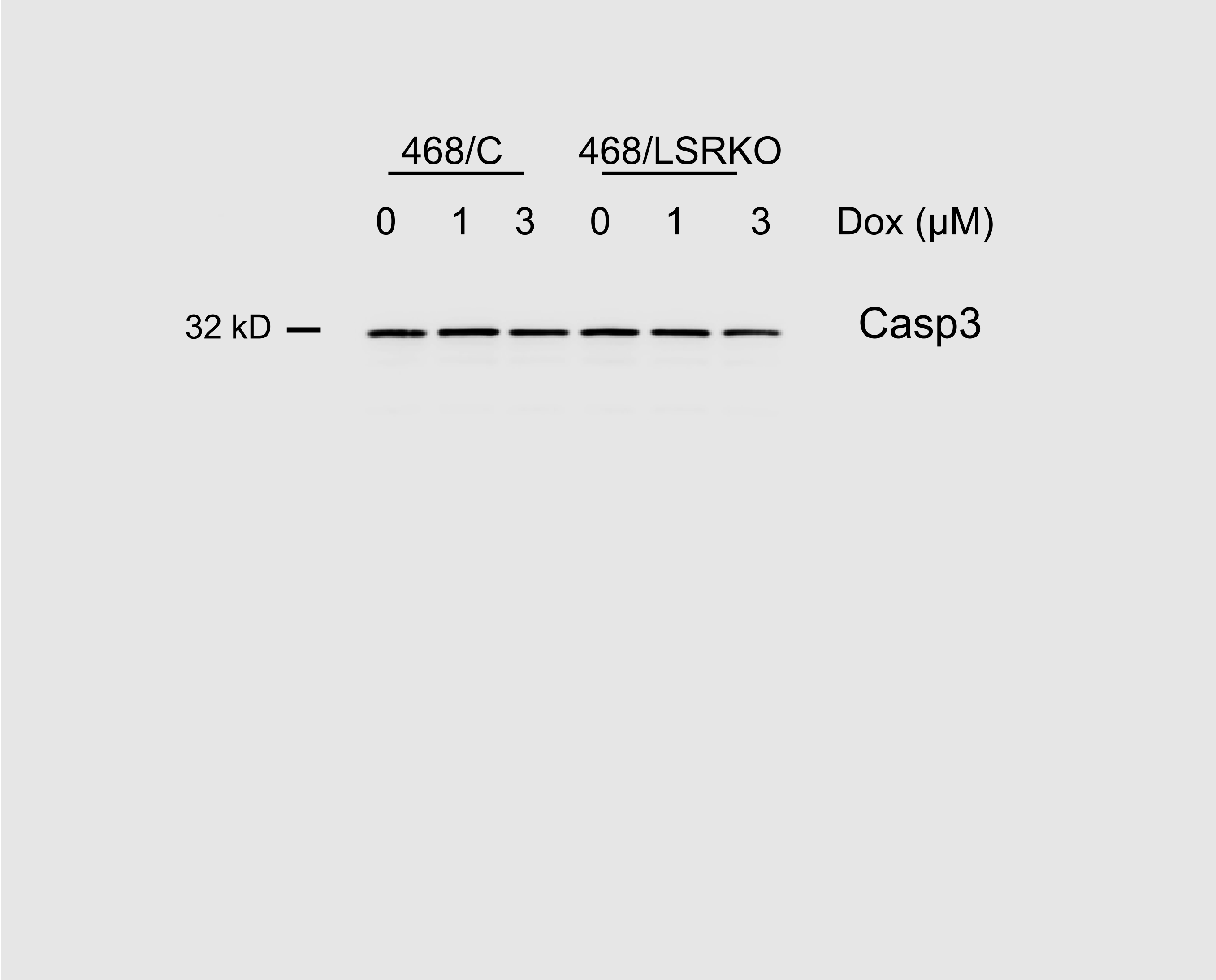

Fig4I-c-Casp3

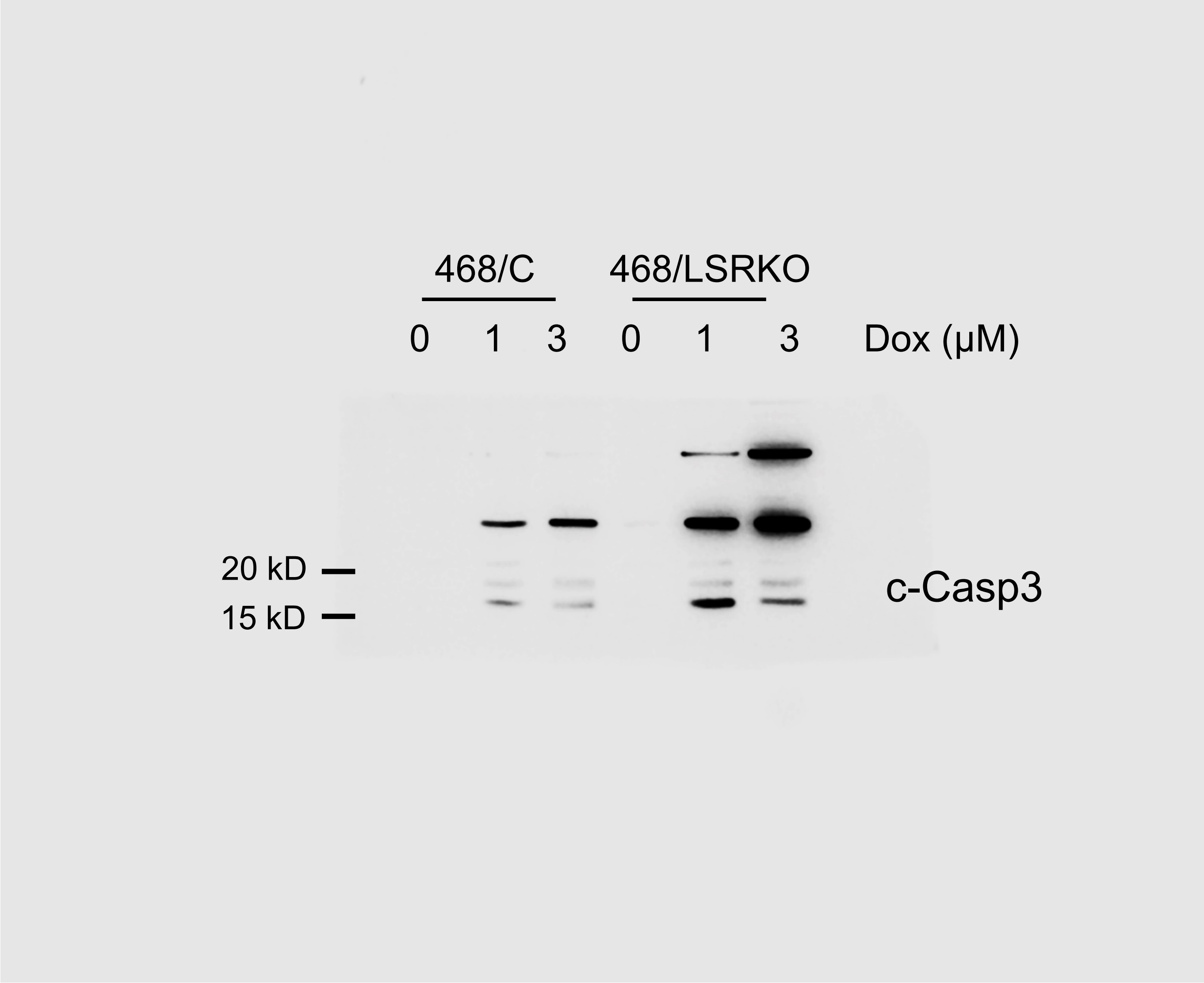

Fig4I-β-Actin

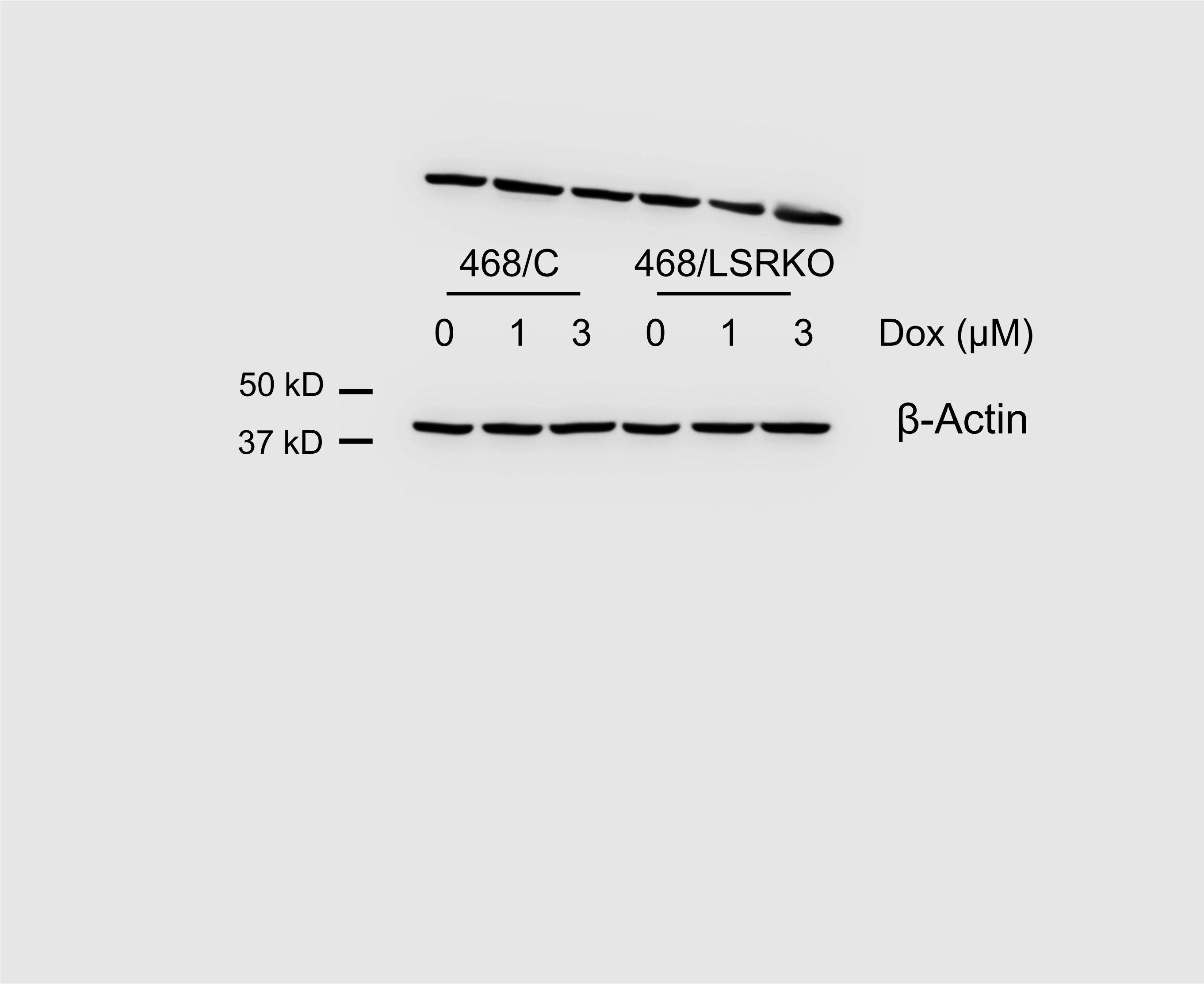

Sup Fig. 1-LSR

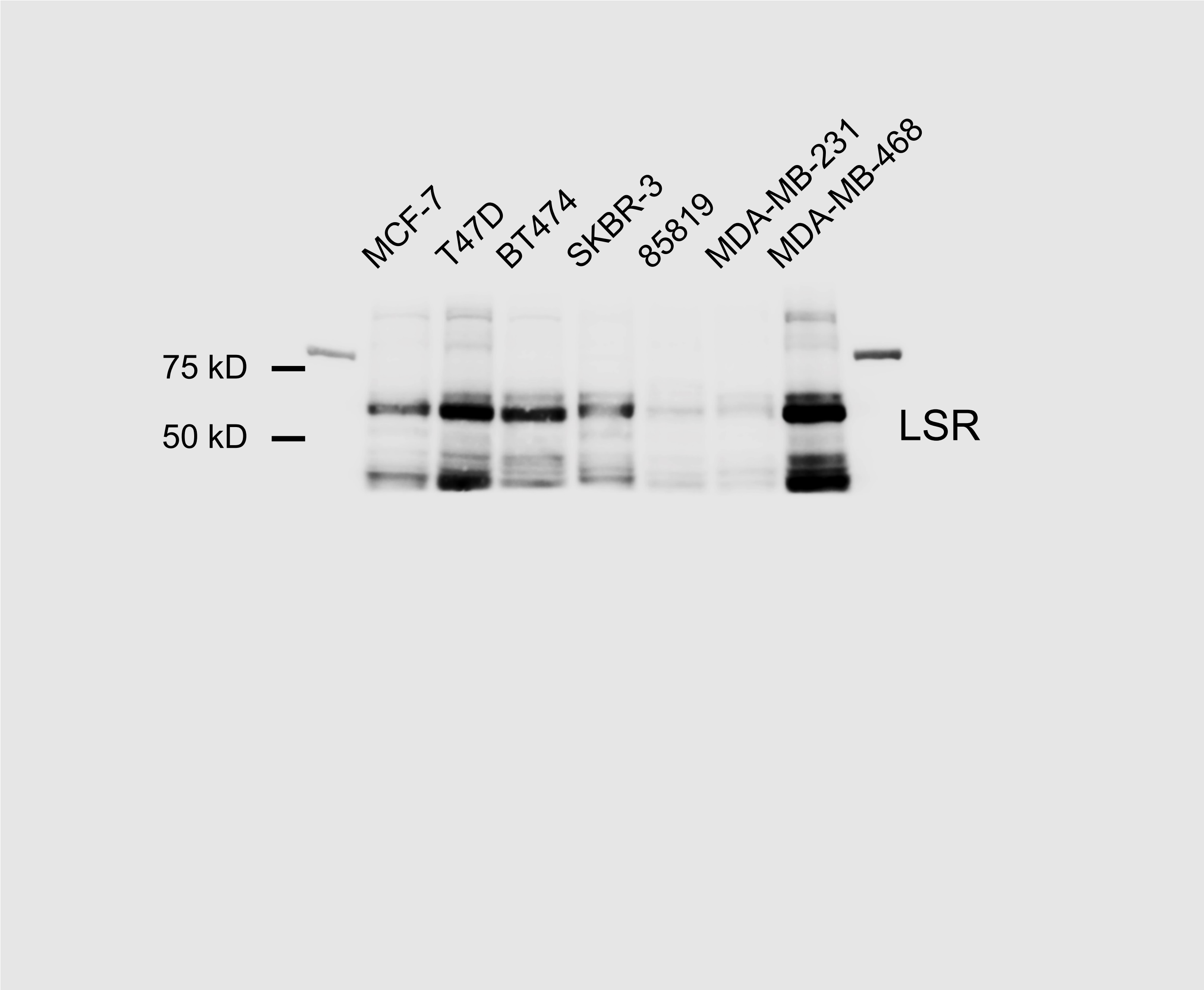
MCF-7
T47D
BT474
SKBR-3
85819
MDA-MB-231
MDA-MB-468
75 kD
50 kD
LSR

Sup Fig1-β-Actin

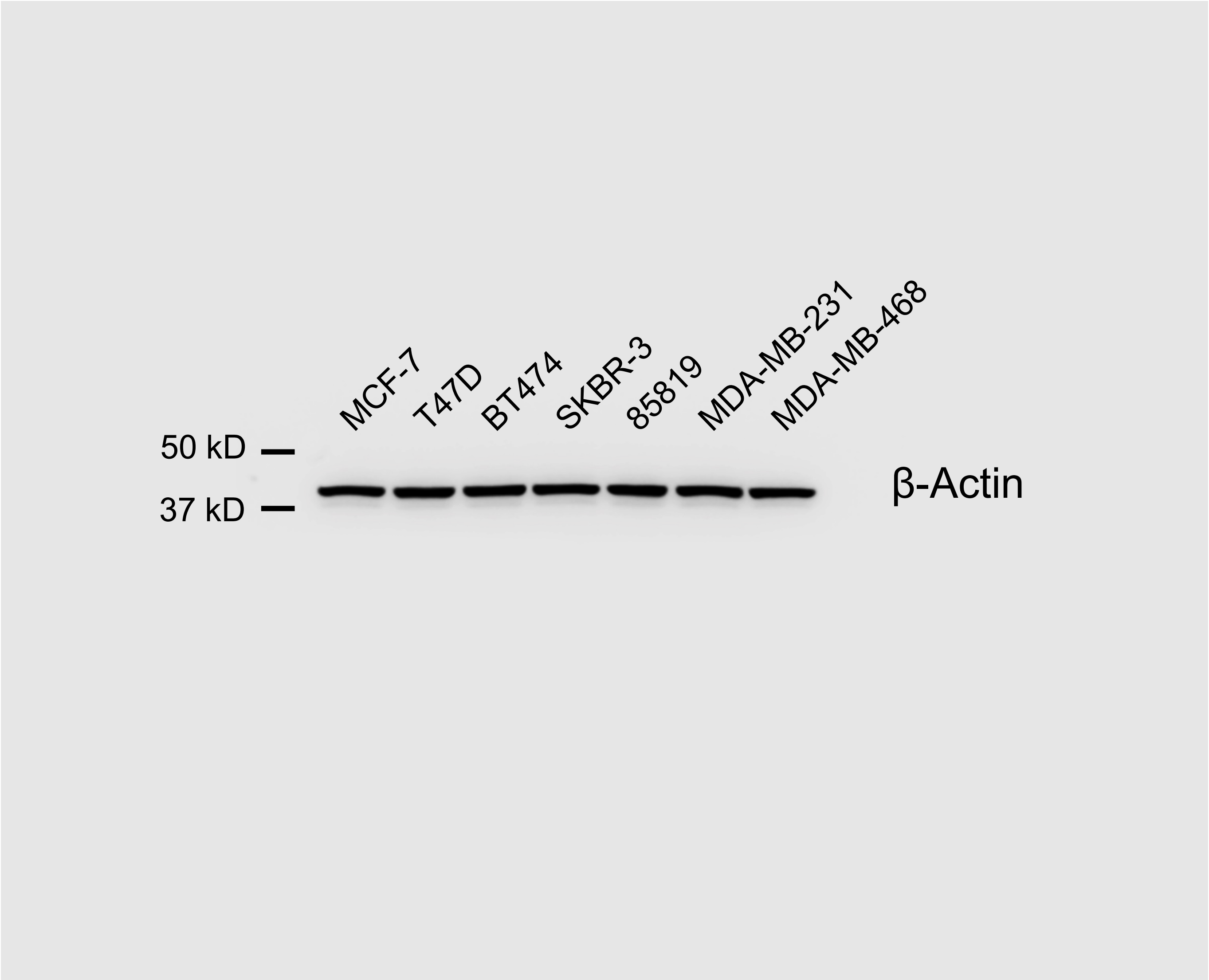

Supplement: S4 Fig — (PDF) [file pone.0336124.s004.pdf]
